# A 90-day oral exposure to food-grade gold at relevant human doses impacts the gut microbiota and the local immune system in a sex-dependent manner in mice

**DOI:** 10.1186/s12989-023-00539-5

**Published:** 2023-07-13

**Authors:** Lauris Evariste, Bruno Lamas, Sandrine Ellero-Simatos, Laure Khoury, Christel Cartier, Eric Gaultier, Benoit Chassaing, Nicolas Feltin, Laurent Devoille, Georges Favre, Marc Audebert, Eric Houdeau

**Affiliations:** 1 Toxalim UMR1331 (Research Centre in Food Toxicology), Toulouse University, INRAE, ENVT, INP-Purpan, UPS, Toulouse, France; 2 PrediTox, Toulouse, France; 3 INSERM U1016, Team “Mucosal Microbiota in Chronic Inflammatory Diseases”, CNRS UMR 8104, Université de Paris, Paris, France; 4 Department of Materials, LNE, Trappes, France

**Keywords:** Edible gold, E175 food additive, Nanoparticles, Microbiota, Intestinal immune response, Sexual dimorphism

## Abstract

**Background:**

Edible gold (Au) is commonly used as a food additive (E175 in EU) for confectionery and cake decorations, coatings and in beverages. Food-grade gold is most often composed of thin Au sheets or flakes exhibiting micro- and nanometric dimensions in their thickness. Concerns about the impact of mineral particles used as food additives on human health are increasing with respect to the particular physico-chemical properties of nanosized particles, which enable them to cross biological barriers and interact with various body cell compartments. In this study, male and female mice were exposed daily to E175 or an Au nanomaterial (Ref-Au) incorporated into food at relevant human dose for 90 days in order to determine the potential toxicity of edible gold.

**Results:**

E175 or Ref-Au exposure in mice did not induce any histomorphological damage of the liver, spleen or intestine, nor any genotoxic effects in the colon and liver despite an apparent higher intestinal absorption level of Au particles in mice exposed to Ref-Au compared to the E175 food additive. No changes in the intestinal microbiota were reported after treatment with Ref-Au, regardless of sex. In contrast, after E175 exposure, an increase in the Firmicutes/Bacteroidetes ratio and in the abundance of Proteobacteria were observed in females, while a decrease in the production of short-chain fatty acids occurred in both sexes. Moreover, increased production of IL-6, TNFα and IL-1β was observed in the colon of female mice at the end of the 90-day exposure to E175, whereas, decreased IL-6, IL-1β, IL-17 and TGFβ levels were found in the male colon.

**Conclusions:**

These results revealed that a 90-day exposure to E175 added to the diet alters the gut microbiota and intestinal immune response in a sex-dependent manner in mice. Within the dose range of human exposure to E175, these alterations remained low in both sexes and mostly appeared to be nontoxic. However, at the higher dose, the observed gut dysbiosis and the intestinal low-grade inflammation in female mice could favour the occurrence of metabolic disorders supporting the establishment of toxic reference values for the safe use of gold as food additive.

**Supplementary Information:**

The online version contains supplementary material available at 10.1186/s12989-023-00539-5.

## Introduction

Gold (Au) is commonly used as a food additive (referred to as E175 in the EU) for the decoration of confectionery, cakes, chocolates and liquors. As with other food additives, such as E171 (titanium dioxide) or E174 (silver), food-grade Au is composed of micro- and/or nanosized particles and is representative of manufactured agents contributing to human daily exposure to nanoparticles through the diet [1–3]. The consumption of products containing E175 leads to a mean daily intake of Au reaching up to 0.31 μg/kg of body weight (BW)/day (d) in children and was estimated to possibly reach up to 1.32 µg/kg BW/d across all population groups [4, 5]. Despite the lack of relevant toxicological data, the consumption of E175 was considered acceptable in 1975 by the Scientific Community for Food, and no acceptable daily intake was determined [5, 6]. There is a rising concern about the potential health risk associated with the presence of nanoparticles (NPs) in food products, as they could exert deleterious effects on health through the alteration of the intestinal flora, gut barrier integrity, and brain development and behaviour [7–11]. These effects are potentially related to the particular physicochemical properties of NPs, which enable them to cross biological barriers such as the intestine, blood–brain barrier and placenta [12–14]. The European Commission requested a scientific opinion on the re-evaluation of the use of edible gold (E175) as a food additive that pointed out the absence of data on E175 chronic toxicity [5]. Indeed, for the majority of the published studies, the focus was on in vitro toxicological effects associated with Au-NP exposure, indicating nonlinear size-dependent cytotoxicity [15], genotoxicity [16, 17] or cellular uptake [18, 19]. On the other hand, due to their potential use for therapeutic applications as nanocarriers [20], most of the in vivo studies conducted emphasized the biodistribution of the compounds, highlighting size-related biodistribution and excretion routes [21–26], with a strong influence of the surface characteristics and administration route [27–29]. Concerning the toxicological effects associated with in vivo exposure to Au-NPs, discrepancies between studies on rodents are noted, with responses ranging from no effect to weight loss, proinflammatory responses and granuloma formation in the liver or high mortality [29–32]. However, neither the nanomodels (100% NPs) nor the administration methods used (e.g., intravenous or intraperitoneal injections) are representative of the food risk encountered by the general population, that is, chronic oral exposure at low doses to a mix of micro- and nanoparticles, as found in the E175 food additive. Indeed, studies of Au-NP toxicity should consider exposure to foodborne Au-NPs, i.e., E175-related, consumed daily by the population through the diet.

Following ingestion, Au-NPs are able to interact with bacteria from the intestinal microbiota [33] before crossing through the gut epithelial barrier [29, 33–35] to interact with local and systemic immune cells [36–38]. The intestinal mucosa represents a complex interface participating in the homeostatic relationship between the gut microbiota and host immune system in response to the environment and diet [39–41]. Indeed, the gut microbiota plays a key role in multiple physiological functions such as energy metabolism, gut immunity and brain development [41–44]. Reciprocally, intestinal epithelial cells and the immune system regulate and shape the gut microbiota [40, 41, 45]. The crosstalk between the microbiota and host cells is complex and involves bacterial metabolites, such as short-chain fatty acids (SCFAs) and aryl hydrocarbon receptor (AhR) ligands, which are essential for the maintenance of intestinal, nervous system and liver functions [46–49]. Many chronic diseases in humans are associated with alterations along the microbiota-immune system axis, such as inflammatory bowel disease and metabolic disorders [50]. Based on the observation that Au-NPs harbour antibacterial activities [51], we hypothesized that a chronic oral exposure to the food-grade Au (E175) could also alter the microbiota-immune system axis, leading to dysbiosis and/or intestinal inflammation with possible health outcomes. Moreover, as sex-related differences have been noticed in the gut microbiota composition and immune responses [52–54], sexual dimorphism could exist with regard to the impacts of E175 on the gut microbiota-immune systems axis, resulting in a sex-related susceptibility to disease development. In this context, the aim of this study in mice was to explore the intestinal and systemic (spleen and liver) biodistribution of the food additive E175 and to determine the potential physiological impact on the digestive tract (intestinal tolerance, including on the gut microbiota) of chronic exposure to E175 at relevant human doses, taking into account a potential sexual dimorphism. Particular attention was given to the size of the particles of the E175 test powder in comparison to a reference (Ref) Au test nanomaterial.

## Results

### Physicochemical characteristics of Au particles

**Fig. 1:**
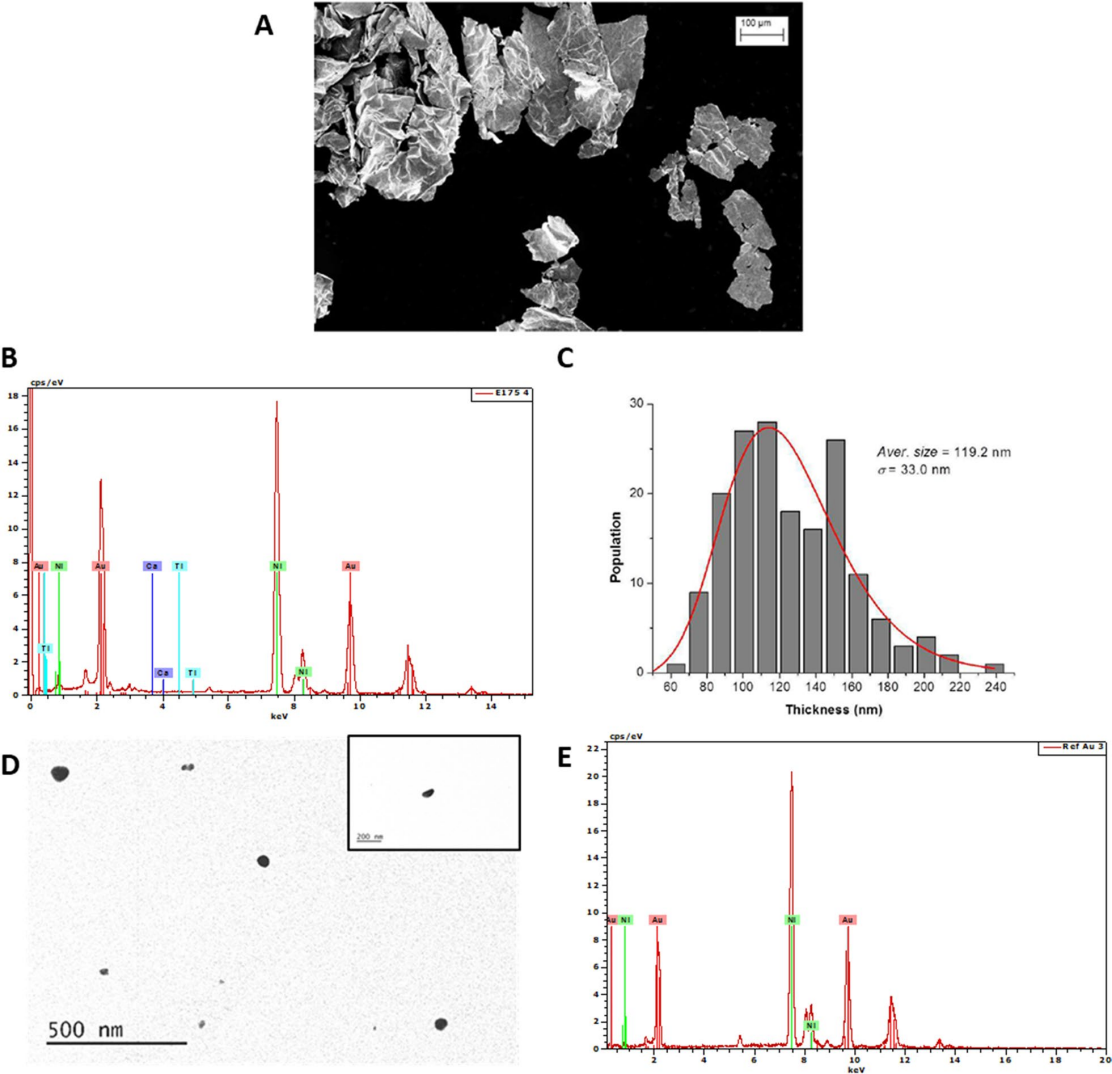
Microscopy analysis of food-grade Au (E175) and Ref-Au particles. **A** Scanning electron microscopy (SEM) image of Au foils in an E175 water suspension. Scale bar = 100 µm. **B** EDX spectra of E175 particulate matter showing Au signals from particles and Ni from the nickel grid. **C** Number distribution histogram of the measured thicknesses of the fragments of Au foils from E175 powder. **D** Transmission electron microscopy (TEM) image of Au particles in a Ref-Au water suspension. Scale bar = 500 nm. **E** EDX spectra of Ref-Au particulate matter showing Au signals from particles and Ni from the nickel grid

Scanning electron microscopy (SEM) and transmission electronic microscopy (TEM) coupled to energy-dispersive X-ray (EDX) analyses demonstrated that the E175 powder was composed of fragments of gold foils, which were close to purity (Fig. 1A, B). The mean thickness of the fragments of gold foils from E175 was 119 ± 8.3 nm with a thickness size distribution ranging from 60 to 240 nm, and 28% of the nano-objects by number were named nanoplates because only one dimension was less than 100 nm (Fig. 1C). According to the manufacturer, the Au-NPs used in this study (Ref-Au) were spherical in shape, and the particle size distribution ranged from 50 to 100 nm. The nanoparticles from Ref-Au were close to purity with a specific surface area of 3.5 m^2^/g (Fig. 1D, E). The nickel peak observed in the EDX spectra of E175 and Ref-Au was from the grid that supported the particles for the microscopy analysis (Fig. 1B, E).

### Animal behaviour, feed consumption and body and organ weights

**Fig. 2:**
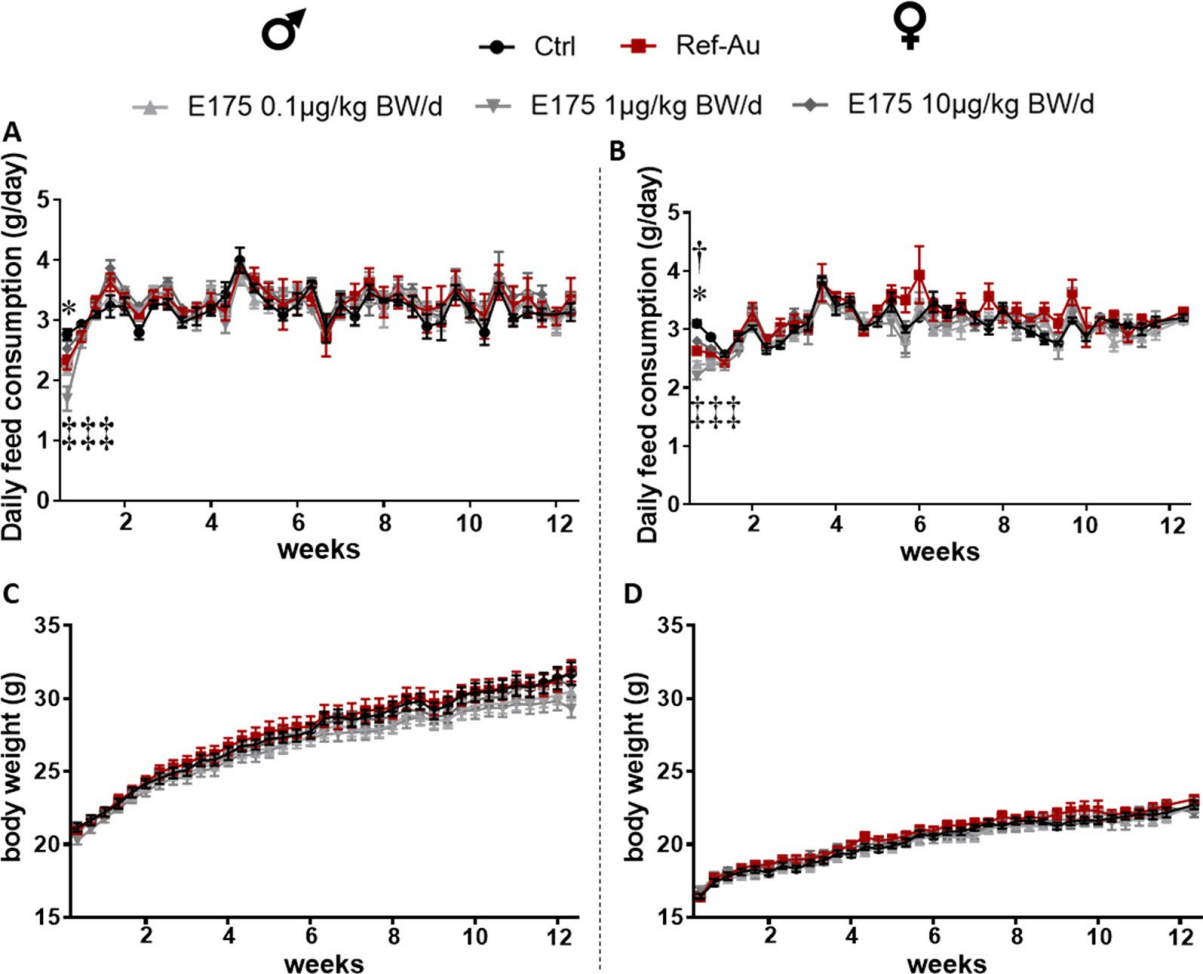
Feed intake and body weight of mice exposed to Ref-Au or E175 for 13 weeks. **A** Daily feed intake of male mice exposed for 13 weeks to Ref-Au at 10 µg/kg BW/d or E175 at 0.1, 1 and 10 µg/kg BW/d incorporated in food pellets. **B** Body weight of male mice orally exposed for 13 weeks to Ref-Au at 10 µg/kg BW/d or E175 at 0.1, 1 and 10 µg/kg BW/d. **C** Daily feed intake of female mice exposed for 13 weeks to Ref-Au at 10 µg/kg BW/d or E175 at 0.1, 1 and 10 µg/kg BW/d incorporated in food pellets. **D** Body weight of female mice orally exposed for 13 weeks to Ref-Au at 10 µg/kg BW/d or E175 at 0.1, 1 and 10 µg/kg BW/d. For statistical comparisons, the dagger (†) indicates Ref-Au versus control, asterisk (*) indicates E175 0.1 µg/kg BW/d versus control, and double dagger (‡) indicates E175 1 µg/kg BW/d versus control. Data are expressed as the mean ± SEM. ^†^P < 0.05; *P < 0.05; ***P < 0.001; ^‡‡‡^P < 0.001; two-way ANOVA and post hoc Tukey’s test

To determine the potential toxicity of food-grade Au (E175), male and female mice were exposed for 13 weeks to E175 incorporated into food pellets at relevant human dose levels of 0.1 and 1 μg/kg BW/d and at a high dose level of 10 μg/kg BW/d. For comparison purposes of the potential size effect of Au particles, groups of male and female mice were treated with a 100% nanosized Au nanomaterial (Ref-Au) incorporated into the food pellets at the higher dose level of 10 μg/kg BW/d. Control male and female mice were fed an untreated diet. During the experiment, all mice appeared healthy, and no abnormal behaviour was observed in mice treated with E175 or Ref-Au. The daily feed intake of male and female mice exposed to E175 or Au-NPs was similar to that of the control group throughout the exposure period except at Day 3 after the beginning of the treatment, where the daily feed intake of male and female mice exposed to E175 at 0.1 and 1 µg/kg BW/d was significantly decreased compared to that of the control mice (Fig. 2A, B). A decrease in daily feed intake was also observed in female mice exposed to Ref-Au compared to the control group at Day 3 after the beginning of the exposure (Fig. 2B). These modulations in the daily feed intake observed only 3 days after the beginning of the treatment had no consequences on the body weights and body weight gain of male and female mice (Fig. 2C, D and Additional file 1: Fig. S1A, B). Absolute liver and spleen weights were also similar between treated and control mice regardless of sex (ANOVA, p > 0.05) (Table 1). The mean exposure doses to E175 and Ref-Au were determined using the recorded pellet consumption and body weights. The feed effective intake was 90% and 110% of the target intake for male and female mice, respectively, showing that the mean exposure doses to E175 and Ref-Au were very close to the target exposure doses (Table S1).

**Table 1:** Body and organ weights in mice exposed to Ref-Au or E175 for 13 weeks

Sex	Group	Body weight at sacrifice (g)	Body weight gain at sacrifice (%)	Liver (g)	Spleen (mg)
Male	Control	31.7 ± 0.8	49.7 ± 2.4	1.43 ± 0.04	81.16 ± 3.50
	Ref-Au 10 µg/kg BW/d	31.9 ± 0.7	52.2 ± 2.8	1.48 ± 0.05	95.55 ± 6.81
	E175 0.1 µg/kg BW/d	30.5 ± 0.7	44.7 ± 2.5	1.40 ± 0.06	78.00 ± 3.08
	E175 1 µg/kg BW/d	29.3 ± 0.6	44.6 ± 1.9	1.37 ± 0.05	75.84 ± 1.63
	E175 10 µg/kg BW/d	30.9 ± 0.8	47.8 ± 3.1	1.38 ± 0.04	74.50 ± 2.51
Female	Control	22.7 ± 0.3	38.3 ± 1.6	1.03 ± 0.02	84.18 ± 3.55
	Ref-Au 10 µg/kg BW/d	23.1 ± 0.2	41.2 ± 1.6	1.06 ± 0.02	81.60 ± 2.91
	E175 0.1 µg/kg BW/d	22.3 ± 0.2	38.4 ± 2.4	1.04 ± 0.04	81.91 ± 4.50
	E175 1 µg/kg BW/d	22.2 ± 0.2	34.5 ± 2	1.05 ± 0.02	76.58 ± 2.81
	E175 10 µg/kg BW/d	22.2 ± 0.3	32.5 ± 1.8	1.01 ± 0.02	82.47 ± 2.22

All data are presented as the mean ± SEM

### Gold tissue distribution

**Fig. 3:**
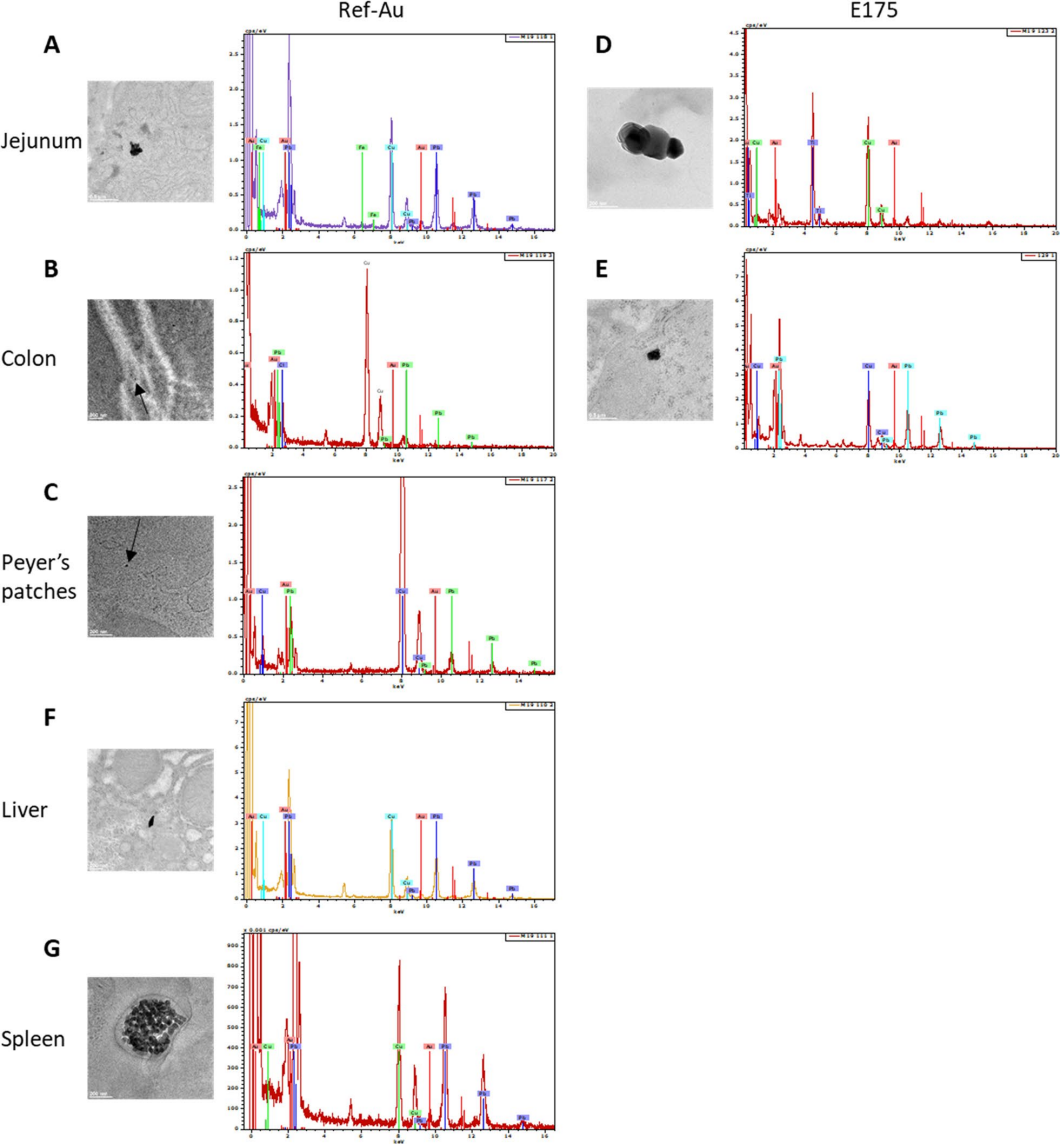
TEM–EDX analysis of digestive or systemic organs in mice exposed to Ref-Au or E175. TEM images of Au particles and their corresponding EDX spectra from jejunum (**A**), colon (**B**), Peyer’s patches (**C**), liver (**F**) and spleen (**G**) of mice orally exposed to Ref-Au (10 µg/kg BW/d) for 13 weeks. TEM images of Au particles and their corresponding EDX spectra from the jejunum (**D**) and colon (**E**) of mice orally exposed to E175 (10 µg/kg BW/d) for 13 weeks

Confocal microscopy for metal particle visualization associated with TEM–EDX analysis was used to monitor Au distribution in the digestive tract, liver and spleen of control mice or mice exposed for 13 weeks to Ref-Au and the highest dose of E175 (10 µg/kg BW/d). While almost no particles were detected in the digestive tract of control mice, the presence of laser-reflecting metal particles was observed in the *lamina propria* of the jejunum villi, in the nuclei of immune cells from the Peyer’s patches and in the colonic lumen from mice fed daily with food containing the Ref-Au nanomaterial (Additional file 1: Figs. S2–4). On TEM tissue sections from mice exposed to Ref-Au, inorganic particulate matter was also observed in the intestinal lumen, in the microvilli and the cytoplasm of jejunum enterocytes as well as in the microvilli of M cells lining the dome of the Peyer’s patch and in colonic epithelial cells (enterocytes and mucus-producing goblet cells) (Additional file 1: Figs. S5–7). Particles were also recovered in areas lining blood vessels in the colonic section (Additional file 1: Fig. S7). Chemical elemental mapping using TEM–EDX confirmed the presence of Au on particle deposits observed in the digestive tract of mice treated with Ref-Au (Fig. 3A–C).

In E175-exposed mice (10 µg/kg BW/d), some laser-reflecting metal particles were observed in the lumen of the jejunum and in the nuclei of immune cells from Peyer’s patches, while no or rare particles were observed in the lumen of the colon (Additional file 1: Figs. S2–4). TEM analysis of tissue sections from mice exposed to E175 showed that electron-dense inorganic particles were found in the intestinal lumen, in the microvilli and in the cytoplasm of jejunum enterocytes as well as close to the nucleus of immune cells in Peyer’s patches and in the nucleus of cells located in the colonic *lamina propria* (Additional file 1: Figs. S8–10). The presence of Au among the particles observed by TEM was confirmed in jejunum and colon tissue sections (Fig. 3D, E). No Au signal was detected in the analysed particles in Peyer’s patches (Additional file 1: Fig. S9). The particles recovered in the Peyer’s patches of mice exposed to E175 were principally composed of silicon (Si) and/or copper (Cu) (Additional file 1: Fig. S9). Interestingly, among the particles analysed by EDX in the digestive tract of mice exposed to Ref-Au or E175, Si- and titanium (Ti)-containing particles were frequently found in addition to Au (Fig. 3D and Additional file 1: Fig. S9).

Gold distribution was also measured in the liver and spleen to determine whether Au particles from the diet containing the Ref-Au nanomaterial or the food-grade gold (E175) reached the systemic circulation and accumulated in these organs. No or rare metal particles were observed in the liver of control and E175-treated mice, while some laser-reflecting metal particles were recovered in the portal space close to the hepatic interlobular artery of mice treated with the Ref-Au nanomaterial (Additional file 1: Fig. S11). Moreover, TEM–EDX analysis confirmed the presence of Au on the particle deposits observed in the liver of mice treated with Ref-Au (Fig. 3F and Additional file 1: Fig. S12). No Au signal was observed in the analysed particles in the liver of mice exposed to E175 (Additional file 1: Fig. S13). The few electron-dense inorganic particles observed in the cytoplasm of hepatocytes of mice exposed to E175 were composed of Cu and calcium (Ca) (Additional file 1: Fig. S13).

In the spleen of control mice, no or few laser-reflecting metal particles were found, while some fluorescent metal particles were observed in the vascular lumen of the splenic artery or translocated inside the splenic tissue of Ref-Au- and E175-treated mice (Additional file 1: Fig. S14). TEM–EDX analysis revealed the presence of few electron-dense inorganic particles with Au signals in the cytoplasm of immune cells in the spleen from Ref-Au-treated mice, while no Au signal was detected in the rare electron-dense inorganic particles found in the spleen of mice exposed to E175 (Fig. 3G and Additional file 1: Figs. S15, 16).

Taken together, these results showed that Au particles translocated in the small and large intestine following 13 weeks of exposure to the Ref-Au nanomaterial or E175. The presence of Au particles in the liver and spleen emphasized a systemic passage of Au nanoparticles from the gut in mice treated with Ref-Au. In contrast, no evidence of systemic passage of Au particles was observed in mice exposed for 13 weeks to a dose of E175 exceeding by tenfold the high exposure level for humans in the maximum exposure level assessment scenario in the EFSA Scientific Opinion of gold (E175) as a food additive.

### Histopathology, genotoxicity and intestinal permeability

**Fig. 4:**
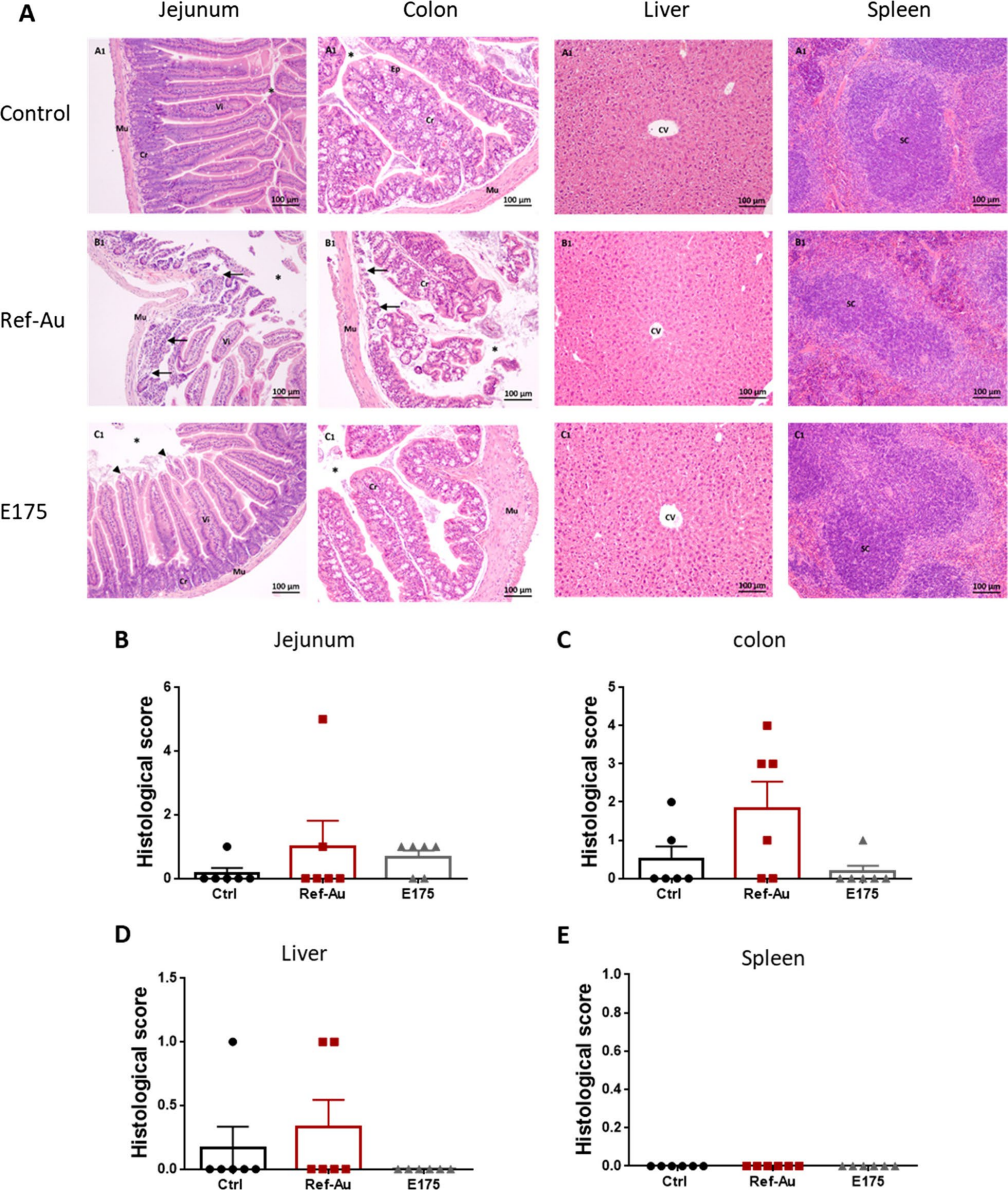
Histological examination of mice organs after exposure to Ref-Au or E175. **A** Haematoxylin–eosin staining of tissue sections from the jejunum, colon, liver and spleen of mice orally exposed to Ref-Au (10 µg/kg BW/d) or E175 (10 µg/kg BW/d) for 13 weeks. Arrows show inflammation of the intestinal mucosa with localized ulceration in the Ref-Au nanomaterial-treated group. In the E175-treated mice, arrowheads show erosion of the upper part of the villi. Magnification ×200. Mu: muscle; Vi: villi; Cr: crypt; Ep: epithelium; *: intestinal lumen; CV: central vein; SC: splenic corpuscle. **B** Histological score in the jejunum, colon, liver and spleen of mice orally exposed to Ref-Au (10 µg/kg BW/d) or E175 (10 µg/kg BW/d) for 13 weeks. Data are expressed as the mean ± SEM. *P < 0.05; one-way ANOVA with post hoc Tukey’s test

Since Au particles were found in the small and large intestines of mice exposed to E175 and Ref-Au as well as in the liver and spleen of mice treated with Ref-Au, histopathological changes in H&E-stained sections, genotoxicity and intestinal permeability were evaluated to determine potential alterations in these organs. No treatment-related histopathological lesions were identified in the jejunum, colon, liver or spleen of Ref-Au- and E175-exposed animals compared to control mice (Fig. 4A–E).

**Fig. 5:**
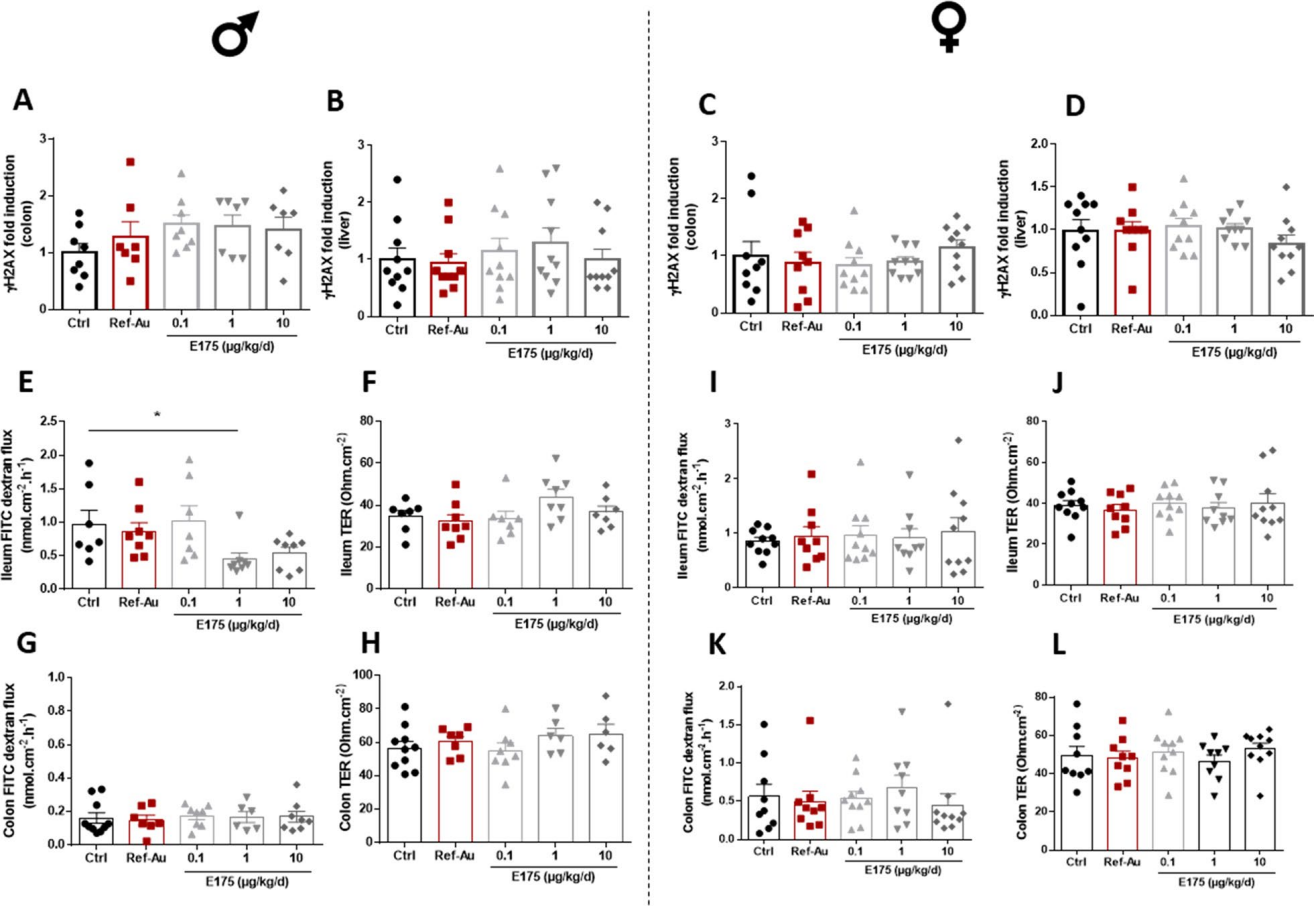
Genotoxicity assessment and intestinal permeability in mice exposed to Ref-Au or E175 for 13 weeks. **A**–**D** Effect of subchronic oral treatment with Ref-Au (10 µg/kg BW/d) and E175 (0.1, 1 and 10 µg/kg BW/d) on γH2AX phosphorylation, a global genotoxic biomarker, in colon (**A**) and liver (**B**) cells of male mice and in colon (**C**) and liver (**D**) cells of female mice. Effect of subchronic oral treatment with Ref-Au (10 µg/kg BW/d) and E175 (0.1; 1 and 10 µg/kg BW/d) on epithelial barrier integrity in the ileum (**E**, **F**) and colon (**G**, **H**) of male mice and in the ileum (**I**, **J**) and colon (**K**, **L**) of female mice. Dextran flux and transepithelial resistance (TER) measurements for 1 h in Ussing chambers in ileum or colon segments from control mice (Ctrl), Ref-Au nanomaterial-treated mice (Ref-Au), and E175-treated mice. FITC dextran: Fluorescein isothiocyanate-dextran. Data are expressed as the mean ± SEM. *P < 0.05; one-way ANOVA with post hoc Tukey’s test

Moreover, the 90-day oral exposure to Ref-Au and E175 did not induce increased phosphorylation of γH2AX, a biomarker of genotoxicity, in the colon and liver of male and female mice (Fig. 5A–D). Likewise, no increase in intestinal permeability nor alteration of epithelial integrity at the end of the Ref-Au or E175 treatment were observed in male and female mice (Fig. 5E–L). Slightly and significantly decreased lumen-to-mucosal permeability of FITC-dextran was noted in the ileum of males treated with E175 at 1 µg/kg BW/d compared to the control group (Fig. 5E). This decrease in the dextran flux was concomitant with an unmodified TER value (Fig. 5F). In addition, the decreased epithelial permeability to FITC-dextran did not occur in a dose-related pattern (similar trend at the high dose level but not significant). These slight changes may be considered to be related to the E175 treatment, but in view of the absence of a dose response (dextran flux and TER) and considering the decreased basal permeability rather than an increase, this effect was not considered deleterious. Altogether, these data demonstrate that the exposure to Ref-Au or E175 does not impair intestinal barrier integrity. In addition, the systemic distribution of gold nanoparticles previously observed is not associated to genotoxic effects in the liver.

### Intestinal immune response

The presence of Au particles in the intestinal tissues of mice exposed to Ref-Au or E175 could suggested that Au particles could interact with intestinal immune cells. To determine whether daily exposure to Ref-Au or E175 impacts the intestinal immune response, we assessed the expression and production of pro- and anti-inflammatory cytokines in the colon of mice. Faecal lipocalin (Lcn)-2 levels, which is used as a general gut inflammatory marker [55], were also evaluated.

Compared to the level in the control group, a dose-dependent trend towards an increase in faecal Lcn-2 levels was noticed in female mice exposed to E175 that did not reach significance (ANOVA, p > 0.05) (Additional file 1: Fig. S17A). Similarly, no differences in faecal Lcn-2 levels were observed in male mice regardless of the treatment considered (Additional file 1: Fig. S17B).

**Fig. 6:**
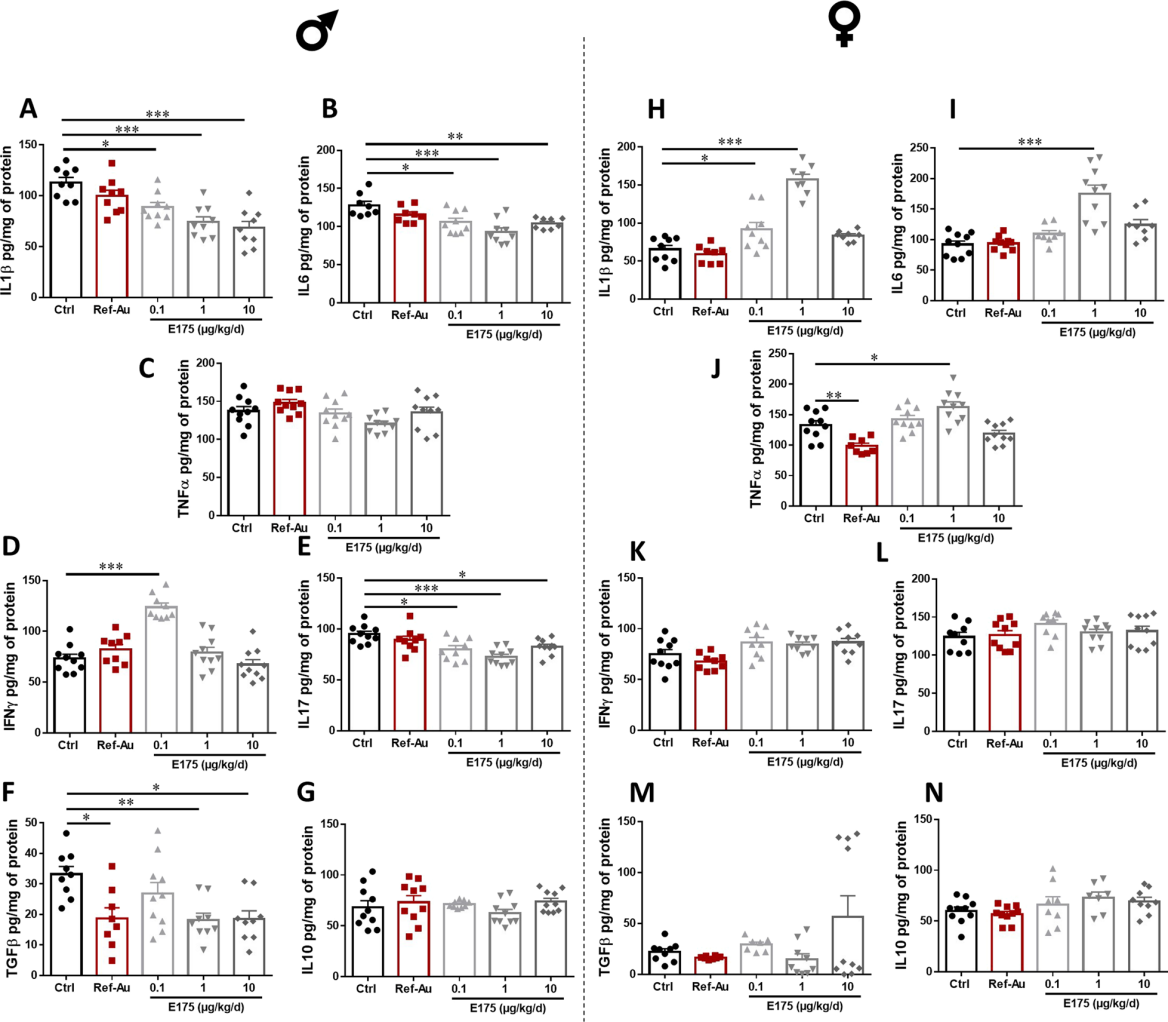
Colonic immune response of male and female mice exposed to Ref-Au or E175. Protein production of the proinflammatory cytokines IL-1β (**A**, **H**), IL-6 (**B**, **I**) TNFα (**C**, **J**), IFNγ (**D**, **K**) and IL-17 (**E**, **L**) as well as of the anti-inflammatory cytokines TGFβ (**F**, **M**) and IL-10 (**G**, **N**) in the colon mucosa of male (**A**–**G**) and female (**H**–**N**) mice orally exposed to Ref-Au (10 µg/kg BW/d) or E175 (0.1, 1 and 10 µg/kg BW/d) for 13 weeks. Each dot represents an individual mouse. Data are presented as the mean ± SEM. Statistical significance was determined by one-way ANOVA with Tukey’s post hoc test or Kruskal–Wallis test followed by Dunn’s post hoc test; *p < 0.05, **p < 0.01, ***p < 0.001

Most notably, decreased production of the proinflammatory cytokines IL-1β, IL-6 and IL-17 was observed in the colon of male mice exposed to all doses of E175 (Fig. 6A, B, E). This decrease in IL-1β levels followed a dose-related pattern, which was not reported for IL-6 and IL-17 (Fig. 6A, B, E). Accordingly, a significant downregulation of the gene expression of IL-1β was noted in male mice exposed to E175 (Additional file 1: Fig. S18A). In contrast, no differences were observed for IL-17 gene expression or for TNFα expression and secretion, regardless of the E175 dose level (Fig. 6C and Additional file 1: Fig. S18B, D). In male mice, the colonic production of the proinflammatory cytokine IFNγ was significantly increased at a dose of 0.1 µg/kg BW/d E175 without a change in the corresponding gene expression (Fig. 6D and Additional file 1: Fig. S18C). A 90-day exposure to E175 also affected the anti-inflammatory response in the colon of male mice through decreased production of the cytokine TGFβ following exposure to the food additive at 1 and 10 µg/kg BW/d (Fig. 6F). No differences occurred for TGFβ gene expression or for the expression and production of the anti-inflammatory cytokine IL-10 regardless of the dose of E175 (Fig. 6G and Additional file 1: Fig. S18E, F). Concerning the impact of a 90-day exposure to Ref-Au in male mice, a significant decrease in IL-1β and IL-10 expression was observed without an effect on protein production (Fig. 6A, G and Additional file 1: Fig. S18A, F). Moreover, decreased production of the cytokine TGFβ was reported in the colon of male mice treated with Ref-Au, while the gene expression of this anti-inflammatory mediator was unchanged (Fig. 6F and Additional file 1: Fig. S18E).

In female mice, a 90-day exposure to Ref-Au led to decreased colonic production of the proinflammatory cytokine TNFα (Fig. 6J). Although a similar decreasing trend was also observed for the gene expression of TNFα in the colon mucosa of female mice exposed to Ref-Au, this effect was not significant (Additional file 1: Fig. S18H). These findings were paralleled by higher IL-6 and TNFα secretion in the colons of female mice exposed to 1 µg/kg BW/d E175 without a change in TNFα expression compared to those of control mice (Fig. 6I, J and Additional file 1: Fig. S18H). In addition, an increase in the secretion of the proinflammatory cytokine IL-1β was reported in female mice exposed to 0.1 and 1 µg/kg BW/d E175, while the IL-1β level did not differ from that of the control group at the highest dose of E175 (Fig. 6H and Additional file 1: Fig. S18G). The gene expression of IL-1β in the colon of female mice followed a dose-related pattern similar to that observed at the protein level, but the increased expression of this proinflammatory cytokine was statistically significant only at the intermediate dose of E175 (Additional file 1: Fig. S18G). Gene expression of the proinflammatory cytokine IL-17 was also significantly increased in female mice exposed to 1 µg/kg BW/d E175 compared to control mice, but there was no change in IL-17 secretion (Fig. 6L and Additional file 1: Fig. S18J). Last, the gene expression and protein production of the anti-inflammatory cytokines IL-10 and TGFβ in the colon mucosa of female mice exposed to E175 were not significantly different from those of control mice (Fig. 6M, N and Additional file 1: Fig. S18K, L).

Altogether, these results showed that 90-day oral exposure to Ref-Au or E175 modulated the intestinal immune response in a sexually dimorphic manner. In male mice, except for an increased IFNγ level only reported at the lowest dose of E175, the production of the proinflammatory cytokines IL-6, IL-1β and IL-17 as well as of the anti-inflammatory cytokine TGFβ was decreased at the end of E175 treatment. In contrast, similar oral exposure to E175 in female mice induced increased production of the proinflammatory cytokines IL-6, TNFα and IL-1β. Moreover, oral exposure to Ref-Au induced a decreased production of TNFα only in female mice. These alterations in the intestinal immune response observed in both sexes were potentially due to the interaction of the Au particles with intestinal microbiota and/or intestinal immune cells.

### Effects of E175 exposure on intestinal AhR activity

**Fig. 7:**
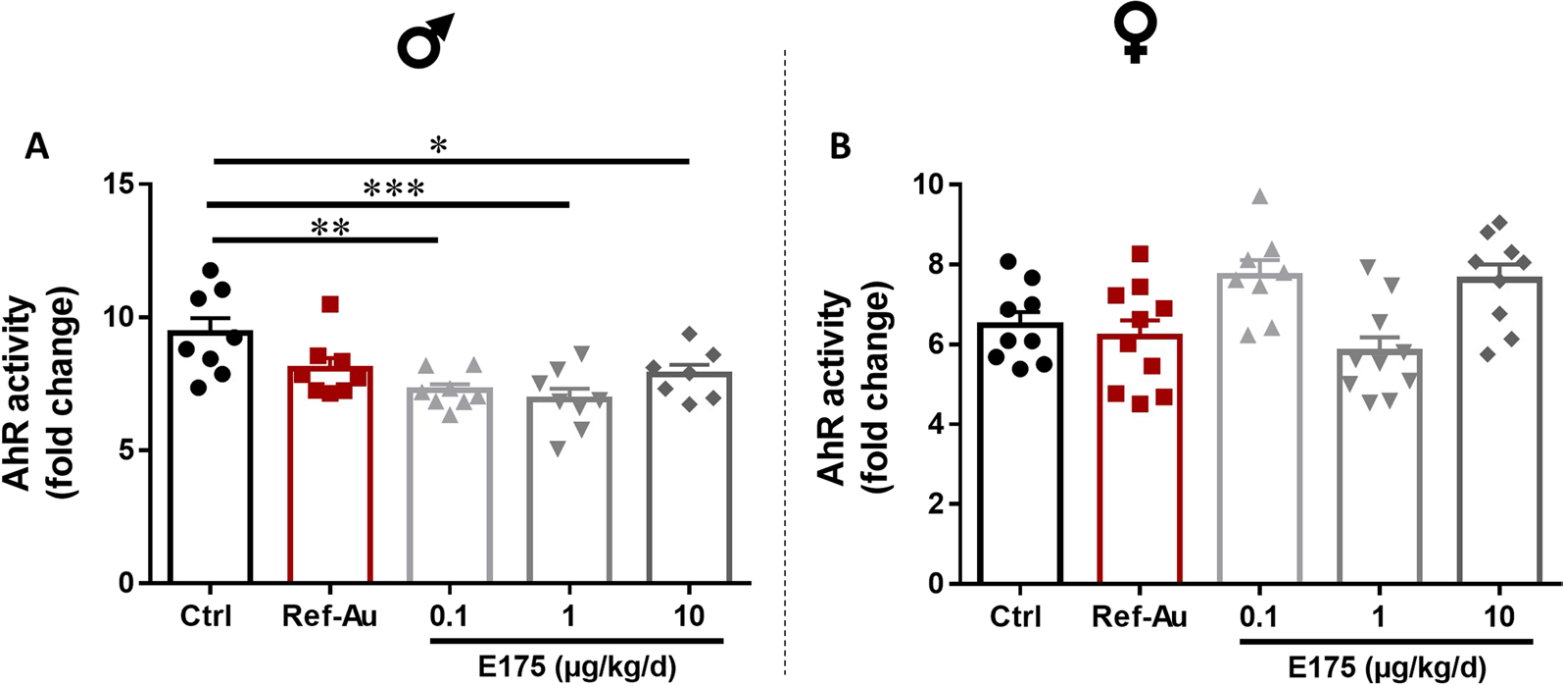
Capacity of faecal microbiota to activate AhR in mice exposed to Ref-Au or E175. Quantification of the faecal AhR activity of **A** male and **B** female mice exposed to Ref-Au (10 µg/kg BW/d) or E175 (0.1, 1 and 10 µg/kg BW/d) for 13 weeks. Data are presented as the mean ± SEM. Statistical significance was determined by one-way ANOVA with Tukey’s post hoc test; *p < 0.05, **p < 0.01, ***p < 0.001

Our results showed that a 90-day exposure to E175 induced decreased production of IL-17 in the colon mucosa of male mice. One mechanism that could link these findings is the modulation of the aryl hydrocarbon receptor (AhR) by the microbiota within the gastrointestinal tract. Indeed, indole derivates generated through the metabolism of tryptophan by the intestinal microbiota have a role in the mucosal immune response via AhR by modulating immune cells that produce IL-17 [56, 57]. Therefore, by examining the activation of AhR by the faecal microbiota, we are able to evaluate the capacity of the microbiota to induce IL-17 production by intestinal immune cells. Using an AhR reporter system, we found that faeces from male mice exposed to E175 were defective in their ability to activate AhR (Fig. 7A). In accordance with the absence of IL-17 modulation observed in the colon mucosa of female mice after E175 or Ref-Au exposure, the capacity of the faecal microbiota to activate AhR was not different in treated female mice compared to the control group (Fig. 7B).

Collectively, these results showed that the decreased production of IL-17 observed in the colon of male mice exposed to E175 could be partly related to the reduced capacity of the faecal microbiota to produce derivates able to activate AhR. This defect in AhR activation by the intestinal microbiota was specifically observed in male mice and confirmed the sexual dimorphism noted in the intestinal production of IL-17 after oral exposure to E175.

### Faecal microbiota composition and function

The systemic absorption of Au-NPs is low in rodents [34, 35] and indicates that the majority of the ingested Au-NPs accumulates in the lumen of the gut, favouring permanent contact of the particles with the intestinal bacteria. In addition, based on the observation that Au-NPs and associated byproducts harbour antibacterial activities [51], chronic oral exposure to Au-NPs or their corresponding food forms could alter the gut microbiota, leading to dysbiosis. Therefore, the impact of subchronic oral exposure to Ref-Au or E175 on faecal microbiota composition and activity was explored.

Principal coordinate analysis revealed a difference in faecal microbiota profiles between male and female mice, regardless of the experimental condition considered (Additional file 1: Fig. S19A). Based on this observation of a sexual dimorphism in intestinal microbiota composition in adult mice, subsequent analysis were conducted on each sex independently.

**Fig. 8:**
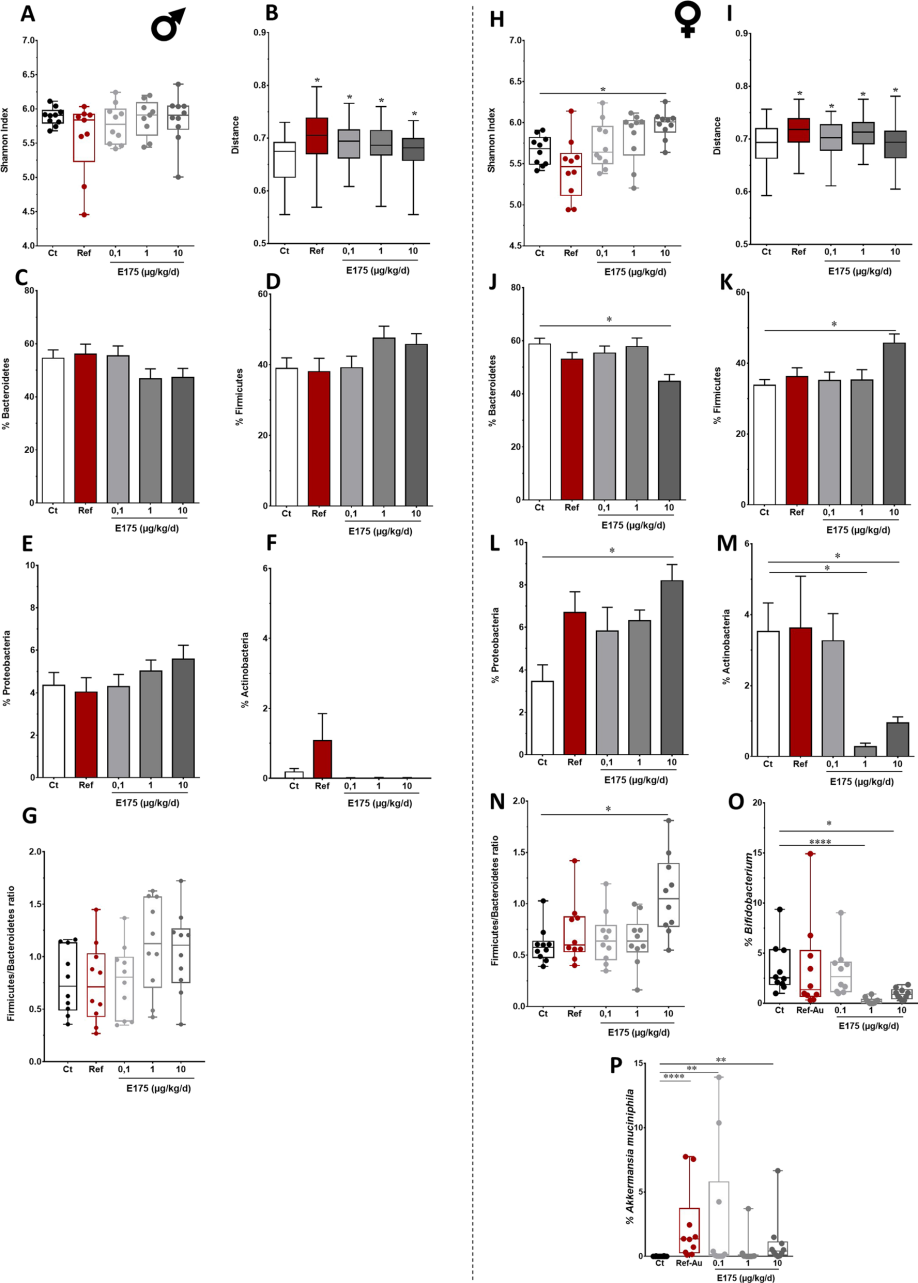
Faecal microbiota composition in male and female mice exposed to Ref-Au or E175. Bacterial diversity based on the Shannon index in the faecal samples from male (**A**) and female (**H**) mice orally exposed to Ref-Au (10 µg/kg BW/d) or E175 (0.1, 1 and 10 µg/kg BW/d) for 13 weeks. Beta diversity based on Bray–Curtis distance in faecal samples from male (**B**) and female (**I**) mice orally exposed to Ref-Au (10 µg/kg BW/d) or E175 (0.1, 1 and 10 µg/kg BW/d) for 13 weeks. Relative abundance of Bacteroidetes, Firmicutes, Proteobacteria and Actinobacteria in faecal samples from male (**C**–**F**) and female (**J**–**M**) mice orally exposed to Ref-Au (10 µg/kg BW/d) or E175 (0.1, 1 and 10 µg/kg BW/d) for 13 weeks. Firmicutes/Bacteroidetes ratio in the faecal microbiota of male (**G**) and female (**N**) mice orally exposed to Ref-Au (10 µg/kg BW/d) or E175 (0.1, 1 and 10 µg/kg BW/d) for 13 weeks. Relative abundance of *Bifidobacterium* (**O**) and *Akkermansia muciniphila* (**P**) in females orally exposed to Ref-Au (10 µg/kg BW/d) or E175 (0.1, 1 and 10 µg/kg BW/d) for 13 weeks. For the Shannon index, differences were statistically tested using ANOVA, followed by Tukey’s test. For beta diversity, PERMANOVA, p < 0.05, followed by pairwise tests were performed. Asterisks indicate conditions that were significantly different from the controls (Ctrl). Changes in phylum relative abundances and the Firmicutes/Bacteroidetes ratio were statistically tested using ANOVA, p < 0.05, followed by Tukey’s test. The relative abundances of the genera *Bifidobacterium* and *Akkermansia* were statistically tested using the Kruskal–Wallis test, as the assumption of a normal distribution of data was not met, P < 0.05, followed by Dunn’s post hoc test

Exposure to Ref-Au did not affect the richness of the gut bacterial communities in male mice, while a significant increase was observed in female mice following exposure to the higher dose of E175 (Fig. 8A, H). In addition, beta diversity analysis in female and male mice using Bray–Curtis distances indicated a significant increase of dissimilarities in bacterial community abundances between the control and the exposed groups regardless of the dose of the treatment (Fig. 8B, I; Additional file 1: Fig. S19B, C).

The faecal microbiota of control and exposed male and female mice was dominated by members of the phyla Firmicutes, Bacteroidetes and Proteobacteria (Additional file 1: Fig. S19D, E). No treatment-related gut microbiota alterations were identified at the phylum or genus levels in exposed male mice (Fig. 8C–G). In female mice, no differences in faecal microbiota composition were observed at the phylum level following exposure to Ref-Au, while the phyla Bacteroidetes and Actinobacteria were significantly decreased after exposure to the highest dose of E175, benefiting to the phyla Firmicutes and Proteobacteria (Fig. 8J–M). As a consequence, a significant increase in the Firmicutes/Bacteroidetes (F/B) ratio was observed following exposure to E175 at 10 µg/kg BW/d (Fig. 8N). Moreover, the abundance of *Bifidobacterium* (genus) was significantly decreased in female mice exposed to E175 at 1 and 10 µg/kg BW/d, and the abundance of *Akkermansia muciniphila* (species) was increased after exposure to Ref-Au and E175 at 1 and 10 µg/kg BW/d (Fig. 8O, P).

To gain insight into the functional differences between the intestinal microbiota from exposed and unexposed male and female mice, we explored the metabolic activity of the faecal microbiota using ^1^H-NMR analysis. This approach allows the detection of 40–50 metabolites, most of which are either produced by gut microbiota metabolism or host-gut microbiota co-metabolism.

**Fig. 9:**
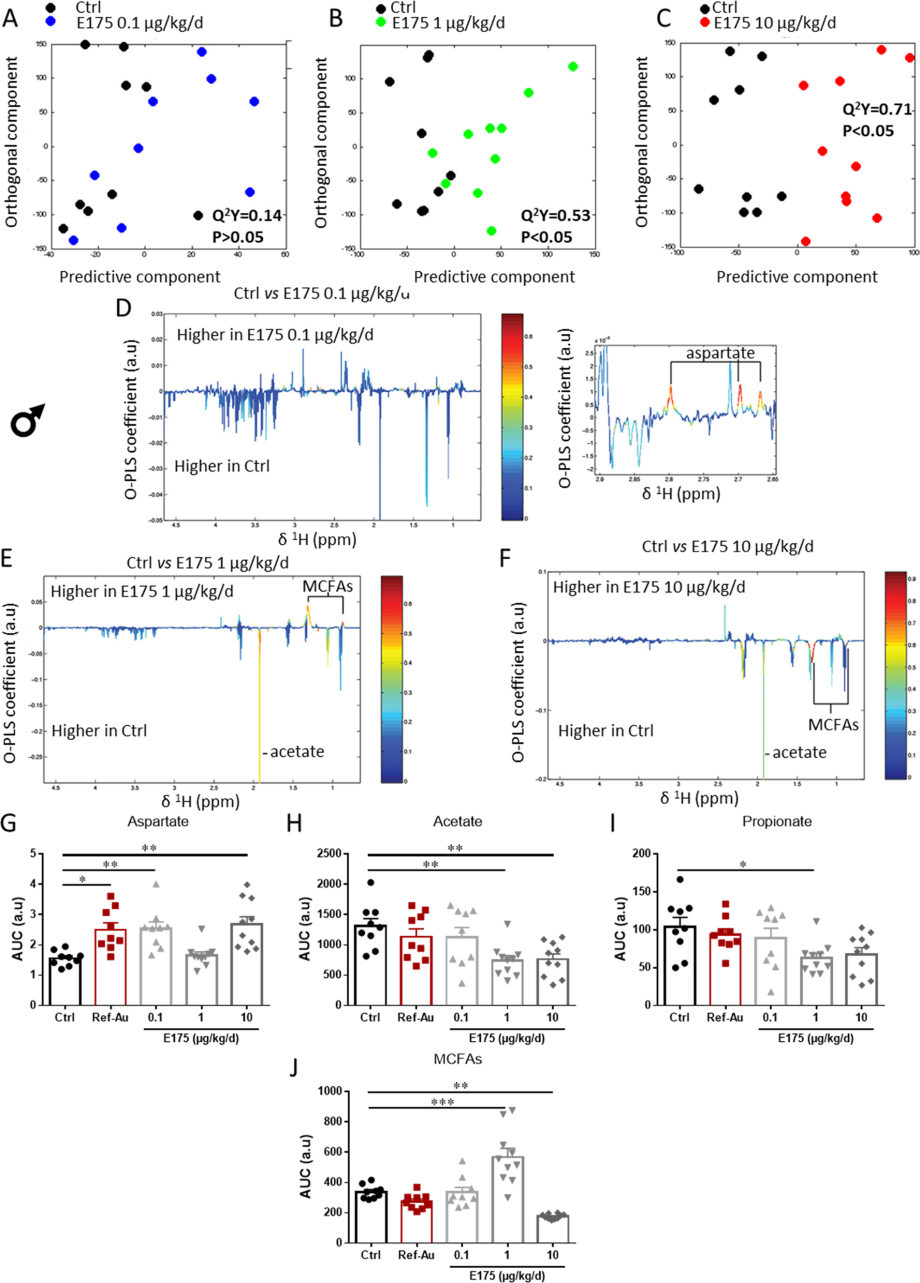
Metabolic activity of the gut microbiota in male mice exposed to Ref-Au or E175. **A**–**C** Orthogonal projection on latent structure-discriminant analysis (O-PLS-DA) score plots derived from ^1^H-NMR spectra of faecal extract from males exposed to E175 at 0.1 (**A**), 1 (**B**) and 10 (**C**) µg/kg BW/day and males exposed to untreated food pellets (Ctrl). **D**–**F** Coefficient plots related to the O-PLS-DA models discriminating between males exposed to E175 at 0.1 (**D**), 1 (**E**) and 10 (**F**) µg/kg BW/day and males exposed to untreated food pellets (Ctrl). The figure shows the discriminant metabolites that are higher or lower in males exposed to E175 at 0.1 (**D**), 1 (**E**) and 10 (**F**) µg/kg BW/day compared to males exposed to untreated food pellets. Metabolites are colour-coded according to their correlation coefficient, with red indicating a very strong positive correlation. The direction of the metabolite indicates the group with which it is positively associated, as labelled on the diagram. **G**–**J** The area under the curve (AUC) of the ^1^H-NMR spectra was integrated for aspartate (**G**), acetate (**H**), propionate (**I**) and medium-chain fatty acid (MCFA) (**J**) signals for male mice exposed to Ref-Au (10 µg/kg BW/d) or E175 (0.1, 1 and 10 µg/kg BW/d) for 13 weeks. Each dot represents an individual mouse. Data from one independent experiment are presented as the mean ± SEM. Statistical significance was determined by one-way ANOVA with Sidak’s post hoc test; *p < 0.05, **p < 0.01, ***p < 0.001

The ^1^H-NMR-based metabolomics showed that a 90-day exposure to Ref-Au induced significant changes in the faecal metabolic profiles in male (Additional file 1: Fig. S20A, B) but not female mice (Additional file 1: Fig. S20C, D). An orthogonal projection on latent structure-discriminant analysis (O-PLS-DA) significantly discriminated males exposed to Ref-Au from control males (O-PLS-DA model: Q^2^Y = 0.42). The metabolic profiles of the Ref-Au nanomaterial-treated male mice were mainly differentiated from the control group by an increased production level of aspartate (Fig. 9G and Additional file 1: Fig. S20B).

**Fig. 10:**
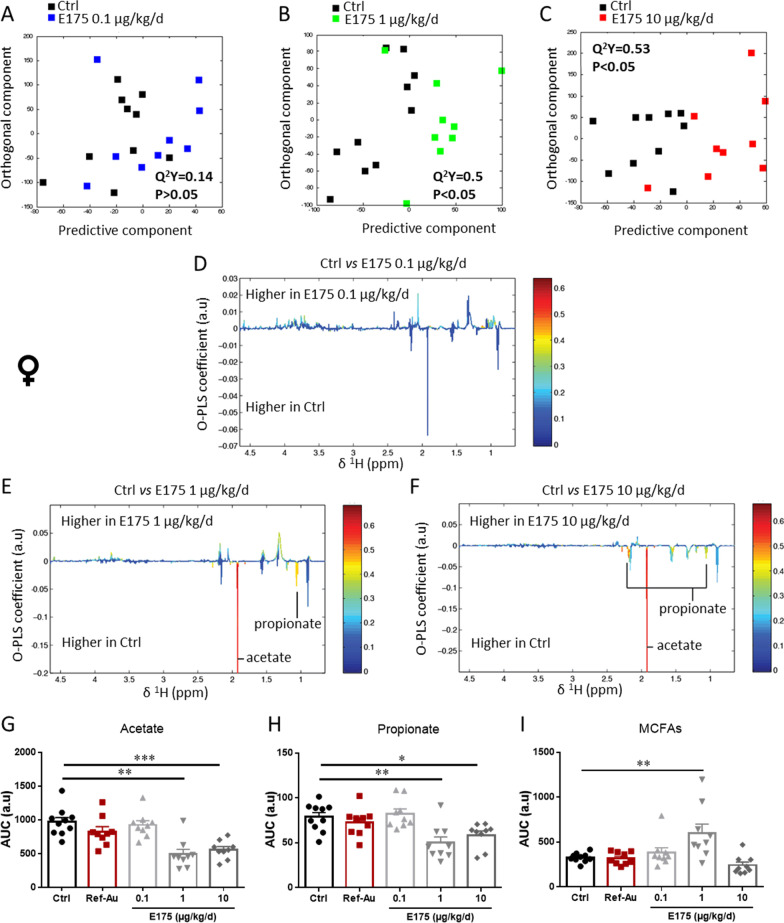
Metabolic activity of the gut microbiota in female mice exposed to Ref-Au or E175. **A**–**C** Orthogonal projection on latent structure-discriminant analysis (O-PLS-DA) score plots derived from ^1^H-NMR spectra of faecal extract from females exposed to E175 at 0.1 (**A**), 1 (**B**) and 10 (**C**) µg/kg BW/day and females exposed to untreated food pellets (Ctrl). **D**–**F** Coefficient plots related to the O-PLS-DA models discriminating between females exposed to E175 at 0.1 **D**, 1 **E** and 10 **F** µg/kg BW/day and females exposed to untreated food pellets (Ctrl). The figure shows the discriminant metabolites that are higher or lower in females exposed to E175 at 0.1, 1 and 10 µg/kg BW/day compared to females exposed to untreated food pellets. Metabolites are colour-coded according to their correlation coefficient, with red indicating a very strong positive correlation. The direction of the metabolite indicates the group with which it is positively associated, as labelled on the diagram. **G**–**I** The area under the curve (AUC) of the ^1^H-NMR spectra was integrated for acetate (**G**), propionate (**H**) and medium-chain fatty acid (MCFA) (**I**) signals for female mice exposed to Ref-Au (10 µg/kg BW/d) or E175 (0.1, 1 and 10 µg/kg BW/d) for 13 weeks. Each dot represents an individual mouse. Data from one independent experiment are presented as the mean ± SEM. Statistical significance was determined by one-way ANOVA with Sidak’s post hoc test; *p < 0.05, **p < 0.01, ***p < 0.001

Following 90 days of exposure to E175 at 0.1 µg/kg BW/d, no significant difference relative to the control group was determined for either sex with the O-PLS-DA statistical model (O-PLS-DA model: Q^2^Y = 0.14) (Figs. 9A and 10A, D). However, the analysis of discriminant metabolites revealed higher levels of aspartate in male mice treated with the low dose of E175 compared to the control group (Fig. 9D, G). In addition, a clear discrimination between the metabolic profiles of the control group compared to male and female mice exposed to 1 and 10 µg/kg BW/d of E175 was observed (Figs. 9B, C and 10B, C). Compared to the control, males exposed to 1 µg/kg BW/d E175 exhibited higher faecal levels of aspartate and medium-chain fatty acids (MCFAs) and lower levels of acetate and propionate, which are both short-chain fatty acids (SCFAs) (Fig. 9E, G–J). Analysis of discriminant metabolites in males exposed to 10 µg/kg BW/d E175 showed higher levels of aspartate and lower levels of acetate and MCFAs than those in unexposed mice (Fig. 9F, G–J).

In female mice exposed to 1 and 10 µg/kg BW/d E175, the metabolic profiles were differentiated from control mice mainly by lower levels of acetate and propionate (Fig. 10B, C, E–I). An increased level of MCFAs was also observed in females exposed to 1 µg/kg BW/d E175 compared to untreated controls (Fig. 10I).

Altogether, these results demonstrate that subchronic exposure to Ref-Au is not sufficient to induce significant alterations of the faecal microbial composition in males and females, suggesting a limited effect of the nanosized Au particles at a high dose level. In contrast, the intestinal microbiota composition and production of metabolites involved in host physiology, such as SCFAs, were altered in male and female mice exposed to E175. These microbiota alterations induced by E175 ingestion were more pronounced in female mice, with a negative shift in the microbial community, favouring the growth of pathogenic bacteria such as Proteobacteria at the expense of beneficial strains such as *Bifidobacterium*.

## Discussion

Edible gold (Au) is composed of mixed nano- and micron-sized thin sheets or flakes of elemental Au [58]. The present study in mice has investigated the intestinal absorption and the potential toxicity of the E175 food additive following exposure to relevant doses for humans. Special attention was given to the particle size-related effects in comparison to a pure Au nanomaterial (herein, Ref-Au, i.e., 100% composed of NPs). To ensure relevance for risk assessment in humans [59], food-grade gold (E175) particles and Ref-Au were incorporated into a solid food matrix (rodent pellets), and mice were exposed for 13 weeks through the diet. No mortality was observed, and body weight evolution appeared to be consistent and similarly increased in all groups, indicating no overt toxicity of the Au-NPs or the E175 additive at the tested doses. In addition, no treatment-related histomorphological damage in the liver, spleen and intestine or genotoxic effects in the colon and liver were observed for either compound at the end of the 13-week oral toxicity study. To date, the few studies on the oral toxicity of Au particles have been carried out in rats or mice using high concentrations of coated or uncoated Au-NPs (20–17,000 µg/kg BW/d) administered in water suspension by gavage for shorter time periods (between 1 and 28 days) [29, 33–35, 60, 61]. Contradictory results were reported, some studies have demonstrated no or low toxicity based on body and organ weights as well as on histopathological, haematological and serum biochemical analysis [29, 33–35, 60], while others have revealed deeper alterations in some of these parameters [29, 37, 61]. Differences in animal species, duration of exposure, dose, size, shape and coating of Au-NPs may contribute to these discrepancies. Furthermore, while in vivo studies in rodents concluded that oral Au-NPs entered the bloodstream and accumulated in the kidney and liver [29, 33–35], these conditions using high dose levels and spherical nanomodels are not representative of E175 exposure in humans, which occurs at very low doses of ingested thin foils or larger sheets, some of which have dimensions < 100 nm in thickness (i.e., nanoplates).

In our study, after thirteen weeks of oral exposure to E175 added to food pellets, Au particles were commonly recovered in the lumen of the jejunum and colon of treated mice, whereas no Au element was evidenced in the liver and spleen. This observation highlighted the almost absent gut-to-blood passage of gold from the food additive, a situation that contrasted with Au-NP treatment herein recovered in the liver and spleen of mice exposed to the Ref-Au. Gold particles were shown to cross the intestinal epithelia in vitro with a size-dependent diffusion capacity, highlighting a higher permeability to Au-NPs than to their microparticulate forms [62]. Accordingly, our in vivo study confirmed the systemic passage of Au-NPs in mice exposed to pure nanosized matter, while no similar translocation was observed in mice exposed to the food additive E175, which was mainly composed of larger Au particles. It is reasonable to conclude that the physical form of edible gold in our batch of E175, where only 28% of the particles were on the order of nanosized in thickness, considerably limits their absorption along the gut. Of note, no significant change in intestinal permeability or food intake was observed in the Ref-Au group of mice, indicating that NP absorption did not result from a loss of epithelial barrier integrity or higher gold consumption during the treatment. On the other hand, the relatively low occurrence of Au signals in the liver of mice exposed to the Ref-Au suggested a limited hepatic retention of NPs after daily exposure for 90 days through food. This is in accordance with a previous biodistribution study of Au-NP models in rats, which demonstrated low systemic absorption with elemental gold mainly eliminated through the faeces [35].

Due to the well-known antibacterial properties of elemental gold [51], it is conceivable that Au accumulation in the lumen of the digestive tract after long-term exposure to E175 could alter the intestinal microbiota, both in its microbial composition and metabolic activity. The gut microbiota plays a crucial role in several vital functions, such as digestive, metabolic and immune functions [50], but this important component of the gastrointestinal tract has received no attention after exposure to edible gold on a chronic basis. In our study, a principal coordinate analysis performed on the overall data indicated that the bacterial community composition significantly differed between male and female mice, regardless of the treatment condition, i.e., including in the untreated (control) groups. This result is in full accordance with previous studies which have reported a sex difference in microbiota composition [52, 53, 63], and this led us to suggest that the impact of Au particles on intestinal microbiota could differ between sexes. Indeed, we reported more significant changes in the intestinal microbiota of female mice exposed to the Ref-Au or the food-grade Au form than in males. In female mice exposed for 13 weeks to Ref-Au, the analysis of the richness and diversity of the bacterial communities in the intestine showed increased diversity in bacterial species with an increased abundance of *Akkermansia muciniphila*. The mucin-degrading bacterium *Akkermansia muciniphila* is recognized as beneficial through local anti-inflammatory effects and has been shown to protect against metabolic disorders such as obesity [64–66]. In a study using male mice exposed orally to a 1700-fold higher dose of Au-NPs, a decreased bacterial richness of the gut microbiota was observed together with an increased abundance of Firmicutes (mainly *Enterococcus*, *Turibacter* and *Lachnospirace*) and a decreased proportion of Bacteroidetes, such as *Bacteroides*, *Alistipes* and *Rickenella* [33]. Interestingly, some of these alterations in the gut microbiota were lost at lower doses of exposure as well as after coating the Au-NPs with 4,6-diamino-2-pyrimidinethiol, showing a coating- and a dose-related effect of the Au-NPs on the gut microbiota [33]. In accordance, we observed a dose-related effect in our study, with more significant changes in the intestinal microbiota in females exposed to the highest dose of E175 (10 µg/kg BW/d). At this dosage and only in females, decreased proportions of Actinobacteria and Bacteroidetes together with increased frequencies of Firmicutes and Proteobacteria led to a significant increase in the F/B ratio. The production of the SCFAs acetate and propionate was also decreased at this dose level in this sex, while at 1 µg/kg BW/d E175, only the decreased abundance of Actinobacteria and the drop in SCFA levels remained significant. Changes in acetate and propionate levels could be linked to the decrease in *Bifidobacterium,* which belongs to the SCFA-producing phylum Actinobacteria [67]. None of these effects were reported at 0.1 µg/kg BW/d of E175, highlighting the dose-related changes in intestinal microbiota composition and activity in female mice. In chronic human diseases associated with gut dysbiosis, such as obesity and overweight [68, 69], an altered F/B ratio is commonly reported [70–72] and helps to predict a decrease in the relative abundance of SCFAs [73, 74]. Furthermore, Proteobacteria are often overrepresented in several intestinal and extra-intestinal diseases with an inflammatory phenotype [75, 76]. Similar F/B ratio and Proteobacteria changes were reported in female mice exposed to E175, suggesting that chronic exposure to high doses of edible gold could increase susceptibility to metabolic disorders. From a toxicological point of view, this emphasizes that the negative and dose-dependent shift linked to E175 exposure on the microbial community in the intestine can be considered a deleterious effect of the food additive. Most of these effects were first reported at a dose (1 µg/kg BW/d) close to dietary levels in humans according to the EFSA Opinion [5], with changes in the F/B ratio and proteobacteria abundance observed at tenfold above the high exposure level in these dietary scenarios. Interestingly, because only minor alterations in the intestinal microbiota occurred in the Ref-Au group of mice, regardless of sex, one may suggest that Au-NPs have very limited effects on the gut microbiome in contrast to larger Au particles in the E175 food additive.

Previous studies have highlighted the existence of anti- or proinflammatory microorganisms in the gut. Indeed, metabolites derived from commensal bacteria (such as MCFAs, SCFAs and AhR ligands) can regulate immune cell functions via indirect as well as direct mechanisms [40, 41, 50, 77, 78]. For example, in the present report, the production defect of MCFAs and SCFAs by intestinal bacteria after 13 weeks of exposure to E175 suggested that local immune functions could be affected in response to E175-evoked gut dysbiosis. On the other hand, the biodistribution analysis of Au-NPs in mice exposed to the Ref-Au also showed that particles were recovered inside the Peyer’s patches along the small intestine, suggesting direct interaction with immune cells. In our study, the inflammatory status of the intestine was first assessed using the faecal levels of Lcn-2, which is a protein mainly secreted in the intestinal lumen by neutrophils that provides a sensitive and broadly dynamic means to noninvasively detect intestinal inflammation in mice [55]. Because faecal Lcn-2 levels did not increase at the end of the 90-day treatment with the gold compounds, we reported that neither the Ref-Au nor the food form E175 at the tested doses induced severe inflammation in the gut in both male and female mice. A deeper analysis of the mucosal immune response, however, revealed decreased production of TNFα or TGFβ in the colon exposed to 10 µg/kg BW/d of Ref-Au regardless of sex. No other significant changes in pro- or anti-inflammatory cytokines have been reported with Ref-Au, suggesting a limited effect of Au-NPs on the immune profile in the intestine in both sexes. Previous studies in male mice indicated that Au-NPs (of similar size to the one used in our study) induced spleen inflammation 7 days after intraperitoneal injection, and smaller particles administered at high doses by oral gavage or intravenously increased reactive oxygen species production and promoted inflammation in the liver or spleen [36–38]. Thus, the impact of Au-NPs on the immune response cannot be fully excluded depending on the dose, the administration route and the target organ considered.

Regarding food additives, no studies have evaluated the effects of chronic ingestion of E175 on the intestinal immune response. We demonstrated that long-term oral exposure to E175 at a human-relevant dose (1 µg/kg BW/d) induced increased production of the proinflammatory cytokines TNFα, IL-1β and IL-6 in the colon mucosa of female mice. These effects were not observed at the high dose of 10 µg/kg BW/d of E175, possibly due to a significant change in the composition of the microbiota reported at this dose, which could disrupt the dialogue between the intestinal flora and immune cells. Furthermore, at the high dose of 10 µg/kg BW/d, the food additive could also modulate the immune response by altering the estrogenic hormonal level in female mice. Indeed, estrogens have been shown to regulate functions of numerous immune cells [79–81] and rat ovarian granulosa cells exposed in vitro to Au-NPs for 1 to 5 h produced higher estrogen compared to unexposed cells, while a significant decrease in estrogen levels was observed after 24 h [82]. Of note, the pro-inflammatory cytokines TNFα, IL-1β and IL-6 are principally secreted by immune cells involved in innate immunity, whereas in the current study, no differences were observed in the proinflammatory cytokine secretion of IFNγ (Th1) and IL-17 (Th17) from adaptive immune cells. Moreover, no significant changes were noticed regarding the production of the anti-inflammatory cytokines IL-10 and TGFβ in the colon that normally increase in the case of mucosal inflammation [83, 84]. Altogether, these data indicated that 13 weeks of exposure to a low dose of E175 relevant for humans (i.e., 1 µg/kg BW/d) activated innate immune cells in the colon of female mice only, leading to low-grade (TNFα-, IL-1β- and IL-6-mediated) inflammation, as evidenced by the low levels of faecal Lcn-2. One may hypothesize that the increased production of TNFα and IL-6 in the female colon could be linked to the decreased SCFA produced by the gut microbiota, which are known to inhibit histone deacetylases involved in the regulation of TNFα and IL-6 secretion by macrophages [85].

In male mice, except for the proinflammatory cytokine IFNγ, which was increased after exposure to the lowest E175 dose tested (0.1 µg/kg BW/d), the colonic production of IL-1β, IL-6 and IL-17 and the anti-inflammatory cytokine TGFβ were significantly decreased after the treatment period. These data clearly show that in males, chronic exposure to E175 at human dietary levels reduced intestinal immune activity, in contrast to females. As E175 exposure affected both the pro- and anti-inflammatory cytokine pathways, this did not result in intestinal inflammation under basal conditions, as indicated by the low levels of faecal Lcn-2. We suggest that the decreased production of IL-17 in the male colon exposed to E175 could be partly due to the impaired ability of the microbiota to catabolize tryptophan into AhR ligands. Indeed, indole derivates, which are tryptophan catabolites generated by the gut microbiota, were identified as activators of AhR and are highly expressed on Th17 and innate lymphoid cells group 3 that produce IL-17 [56]. Any modification in AhR ligand production by the microbiota impacts IL-17 levels and therefore acts on the fragile equilibrium between the microbiota and the host cells. Accordingly, we reported in the present study that the capacity of the gut microbiota to activate AhR was decreased following E175 exposure. Furthermore, this defect in AhR activation was observed in male mice only, which confirmed the sexual dimorphism noted in the intestinal production of IL-17 after long-term exposure to the food additive. It has been shown that IL-17 plays a protective role against muco-epithelial bacterial infection [86], which suggests that male mice exposed daily to E175 could be more susceptible to bacterial pathogens due to the drop in IL-17 in the gut mucosa. Interestingly, similar sex-related differences in inflammation profiles have been reported following exposure to polyethylene glycol-coated Au-NPs [87], where a significant increase in the spleen index, an indicator of immune response activation, was demonstrated in females but not in males. Different studies showed that peripheral innate and adaptive immune responses are stronger in females than in males [88, 89], while a reduced innate immune arm and an enhanced adaptive immune response have been observed in the gut of female mice as compared with males [53, 90]. Indeed, studies in mice showed that gene related to inflammatory response and T cell functions were upregulated in colon of female mice [53]. In addition, females exhibited higher percentage of T cells in mesenteric lymph nodes or Peyer’s Patches [53, 90]. A lower abundance of natural killer and of CD80^+^ dendritic cells was also observed in Peyer’s Patches of female [90]. As reported in mice, transcriptomic analysis of Human gut mucosa showed higher expression of gene linked to immune activation and inflammation in women compared to men [91]. This sexual dimorphism in gut mucosal gene expression was associated with an increased level and activation of CD4^+^ T cells in the *lamina propria* of intestine women [91]. These sex differences in intestinal and peripheral immunity possibly might explain differences in microbiota composition between the sexes [52–54] and participate to the sex bias in many immunological diseases in humans, since the higher immune reactivity in women may contribute to their higher risk of developing autoimmune diseases and their greater resistance to various infections compared with men. Overall, this could explain the sex differences reported here, with the gut microbiota-immune systems axis being more affected in female than in male mice when chronically exposed to Ref-Au or the food additive E175 through the diet. It should be noted that in both sexes, the effects of E175 on the intestinal microbiota and/or the intestinal immune response began at doses within the range of dietary exposure levels in humans and were found to be mostly non-toxic to the host.

## Conclusions

Our study reports that a 90-day E175 exposure in mice at human dietary levels from a solid matrix neither induces histomorphological damage in the liver, spleen and intestine nor genotoxic effects in the colon and liver. A higher systemic passage of Au particles was observed in mice exposed to Ref-Au compared with the food additive E175. This suggested a size-dependent absorption of Au particles in the gut, E175 being manufactured in the form of gold flakes after a process of grinding thin foils, a final form called nanoplates which seems to limit their absorption by the intestine. However, due to the very low absorption level, which is in line with previous studies, we concluded that regardless of their size, most ingested Au particles accumulate in the intestinal lumen, where they can modulate the intricate dialogue between the gut microbiota and intestinal immune cells. After 13 weeks of exposure to E175, a more marked alteration of the gut microbiota composition and activity, characterized by an increased F/B ratio and Proteobacteria abundance as well as a decreased production of SCFAs, was noticed in female mice compared to males. These gut microbiota alterations in E175-exposed mice, when associated with low-grade inflammation, as herein shown in the colon of females, could promote the worsening of metabolic disorders in these individuals, for example, under an unbalanced diet. Considering this potential hazard to human health and the use *ad quatum satis* of the E175 in the food industry, the establishment of toxic reference values for the safe use of gold as food additive (E175) should be considered in the human diet.

## Material and methods

### Gold reference nanoparticle and E175 characterization

The E175 sample was obtained from a French commercial supplier of food colouring (OR A DECOR, La Chapelle Rambaud, France) as a representative batch of edible gold used as a food additive in the EU [5]. Its production was obtained from thin gold sheets that were then reduced by a milling process to gold flakes for commercial use as food additives. The reference Au test nanomaterial (Ref-Au) was purchased from SkySpring Nanomaterials Inc. (Houston, Texas, USA) and ranged in size from 50 to 100 nm. The dimensional properties of the E175 sample were analysed by scanning electron microscopy (SEM). The E175 sample preparation protocol for SEM measurements was as follows: a quantity of powder (approximately 10 mg) was mixed with 5 mL of acetone, and the obtained suspension was homogenized with a vortex mixer. Then, a drop of the suspension was deposited on a silicon substrate by speed-coating. This deposition protocol comprises two phases: (1) spreading a drop of suspension over the silicon substrate with a low rotation speed and (2) rapid drying of the drop at a high rotation speed. The SEM equipment used was a Zeiss UltraPlus combining a high-tech Gemini FEG (Field Emission Gun) column with an in-lens detector located within the column (LNE, Trappes, France). The sample consists of gold foils with lateral dimensions ranging from several tens to hundreds of micrometres. In the present study, the accurate E175 dimension measured by SEM corresponds to the thickness of these sheets. Thickness measurements were performed on fragments of foils in several images. The method used was as follows: (1) different fragments of foils with apparent edges were selected and imaged; (2) measurements of thickness were performed on several points of the edge, with these points being regularly spaced; and (3) histograms of the number-based size distribution were constructed from the thickness measurements (172 thickness measurements were performed on different fragments of foils to construct the histogram). The measurements were carried out with the PlatypusTM software platform from Pollen Metrology. Chemical elemental analysis of the E175 and Ref-Au powders was performed by transmission electronic microscopy (TEM) coupled to energy-dispersive X-ray (EDX) spectroscopy. The powders were diluted in water, and a few drops were deposited either on a copper membrane grid for TEM observation or on a nickel grid for EDX analysis. The sections were observed under a JEOL JEM-1400 electron microscope (MeTi facility, Toulouse, France) for TEM observations and were analysed by EDX under a JEOL 2100F for chemical elemental analysis (Raimond Castaing platform, Toulouse, France).

### Animals and treatments

Four-week-old male (M) and female (F) C57BL/6J mice were purchased from Janvier (France) and acclimated for 1 week before use. Throughout the study, mice were maintained in polysulfone cages in a pathogen-free environment maintained at 22 ± 2 °C under a 12-h light–dark cycle. The experimental design was based on a repeated dose (90-day) oral toxicity OECD study according to the guidance on risk assessment of nanomaterials in the food and feed chains [59]. Mice were randomly assigned to experimental groups (n = 10/sex/condition) and fed ad libitum for 13 weeks with untreated diet (0 μg/kg BW/d) or with the E175 food additive incorporated into the food pellets at relevant human dose levels of 0.1 and 1 μg/kg BW/d and at a high dose level of 10 μg/kg BW/d. One supplemental group of mice (n = 10 animals/sex) was treated for 13 weeks with a Ref-Au nanomaterial (100% nanosized, incorporated into the food pellets) at the higher dose level of 10 μg/kg BW/d for comparison of the potential size effect of Au particles. Mice were weighed every two or three days, and food consumption was recorded per cage to calculate the actual E175 or Ref-Au daily intake.

### Examination of particle absorption by confocal microscopy

Tissue samples were embedded in paraffin wax and sectioned at a thickness of approximately 5 µm. Tissue sections were incubated with WGA-Alexa 594 for 1 h in the dark and then washed before being mounted in ProLong Gold antifade mounting medium (Life Technologies, France) containing DAPI (4′,6-diamidino-2-phenylindole) for fluorescence microscopy. Slides were viewed using a Leica SP8 confocal microscope for laser reflection particle detection with the 40 × immersion objective as described previously [13]. Briefly, tissue sections were examined at 488/BP 488–494 nm to detect laser reflection by metal particles and at 514/BP 560–660 nm to visualize WGA staining in the tissue. Inorganic (mainly metal) particles appeared in green (laser reflection), cell nuclei appeared in blue (DAPI staining), and glycosylated parts of cell membranes and mucus cells appeared in red (WGA-Alexa 594 staining).

### Transmission electron microscopy and EDX analysis

Tissue samples were embedded in Spurr resin as described previously [13]. The samples in resin blocks were cut with a Leica ultramicrotome, and 80-nm-thick sections were deposited on copper grids and then stained with a UAR-EMS (Uranyl Acetate Replacement) solution followed by a 0.4% lead citrate solution. The sections were observed under an electron microscope operated at 200 kV using a JEOL JEM-1400 electron microscope (MeTi facility, Toulouse, France) for transmission electronic microscopy (TEM) observations and were analysed by energy-dispersive X-ray spectroscopy (EDX) under a JEOL 2100F for chemical elemental analysis (Raimond Castaing platform, Toulouse, France).

### Ussing chamber experiments

Following mouse euthanasia, ileum and colon segments were used to assess barrier function as described previously [13]. Intestinal strips were mounted in Ussing chambers (Physiological Instruments) with an aperture size of 0.125 cm^2^ and bathed in 1 ml of circulating oxygenated Krebs buffer at 37 °C. Ag–AgCl electrodes were used as short-circuiting electrodes and to measure the transepithelial electrical properties of the tissues throughout the experiment, including the transepithelial potential difference, short-circuit current, and transepithelial resistance (TER), permitting the assessment of tissue viability. After 20 min of equilibration, the paracellular permeability was assessed by measuring the mucosal to serosal flux of FITC-labelled 4-kDa dextran (Sigma) for 1 h, and the results are expressed as the flux of dextran crossing 1 cm^2^ of epithelium per hour (nmol cm^−2^ h^−1^).

### Histology

Collected samples were fixed in 4% formalin for 24 h prior to paraffin embedding. The 5-µm sections were stained with haematoxylin–eosin and examined using light microscopy (90i Nikon microscope). Scoring was blindly performed following the method for the general assessment of intestinal and colonic inflammation that has been previously described [92]. The scoring is based on the visual quantification of inflammatory cell infiltration and the determination of epithelial alteration levels and mucosal structures. For the liver and spleen, histomorphological changes were recorded as previously described [93].

### Cytokine quantification

Colon segments of approximatively 2 cm were placed in Lysing Matrix Tubes (MP Biomedicals) containing 1.4 mm-diameter ceramic spheres and 1 mL of RIPA buffer (1% Igepa, 0.5% deoxycholic acid and 0.1% SDS in Tris buffered saline solution 1×; pH 7.4) with protease inhibitors (Roche Diagnostics). The tubes were shaken at 6 m/s two times for 20 s each in a FastPrep (MP Biomedicals). After centrifugation at 10,000×*g* for 4 min (4 °C), the supernatants were recovered and protein concentrations were assessed using a Coomassie protein assay kit (Thermo Scientific) according to the manufacturer’s instructions. All supernatants were normalized at 1 mg/mL of protein with PBS and stored at − 80 °C until processing. ELISAs were performed on the supernatants to quantify the following mouse cytokines according to the manufacturer’s instructions: IL-10, IL-17A, TNFα and IFNγ (Mabtech, Nacka Strand, Sweden); IL-6, IL-1β and TGFβ (R&D Systems, Minneapolis, MN, USA).

### Quantification of faecal lipocalin-2 levels

Frozen faecal samples were weighed and suspended in cold PBS to determine the faecal lipocalin-2 (Lcn-2) level using a DuoSet murine LCN2 ELISA kit (R&D Systems) as described previously [94].

### Gene expression analysis

Total RNA was isolated from colon samples using a RNeasy Mini Kit (Qiagen) according to the manufacturer’s instructions. Quantitative RT–PCR was performed using SuperScript II Reverse Transcriptase (Life Technologies, Saint Aubin, France) and then a Takyon SYBR Green PCR kit (Eurogentec, Liège, Belgium) with specific mouse oligonucleotides. The primers used were as follows: Gapdh (sense) 5′-AACTTTGGCATTGTGGAAGG-3′, (antisense) 5′-ACACATTGGGGGTAGGAACA-3′; IL-1β (sense) 5′-GCCCATCCTCTGTGACTCAT-3′, (antisense) 5′-AGGCCACAGGTATTTTGTCG-3′; IL-17 (sense) 5′-TTTAACTCCCTTGGCGCA-3′, (antisense) 5′-CTTTCCCTCCGCATTGACAC-3′; TNFα (sense) 5′-TCCCCAAAGGGATGAGAAGTTC-3′, (antisense) 5′-GCGCTGGCTCAGCCACT-3′; IFNγ (sense) 5′-ATGAACGCTACACACTGCATC-3′, (antisense) 5′-CCATCCTTTTGCCAGTTCCTC-3′; IL-10 (sense) 5′-AGAAGCATGGCCCAGAAATCA-3′, (antisense) 5′-GGCCTTGTAGACACCTTGGT-3′; and TGFβ (sense) 5′-ACTGGAGTTGTACGGCAGTG-3′, (antisense) 5′-GGATCCACTTCCAACCCAGG-3′. We used the 2^−ΔΔCt^ quantification method with mouse Gapdh as an endogenous control and the group fed with untreated diet as a calibrator.

### Gut microbiota composition analysis

Faecal DNA was extracted from weighted stool samples according to [40]. Briefly, faecal samples were resuspended for 10 min in 250 μl of 4 M guanidine thiocyanate in 0.1 M Tris (pH 7.5) (Sigma–Aldrich) and 40 μl of 10% N-lauroyl sarcosine (Sigma–Aldrich). After the addition of 500 μl of 5% N-lauroyl sarcosine in 0.1 M phosphate buffer (pH 8.0), the samples were incubated at 70 °C for 1 h. Sterilized silica beads were added, and the tube was shaken at 6.5 m/s three times for 30 s each in a FastPrep (MP Biomedicals) apparatus. Then, polyvinylpolypyrrolidone (15 mg) was added to the tube prior to centrifugation (5 min at 20,000×*g*). After recovery of the supernatant, the pellets were washed with TENP (50 mM Tris (pH 8), 20 mM EDTA (pH 8), 100 mM NaCl, and 1% polyvinylpolypyrrolidone). The new supernatant was added to the first supernatant. The washing step was repeated two times. Nucleic acids were precipitated by the addition of isopropanol and centrifugation (10 min at 20,000×*g*). Pellets were resuspended in 450 μl of 100 mM phosphate buffer, pH 8, and 50 µl of 5 M potassium acetate. The tube was placed on ice overnight and centrifuged at 20,000 × *g* for 30 min. The supernatant was then transferred to a new tube containing 20 μl of RNase (1 mg/ml) and incubated at 37 °C for 30 min. Nucleic acids were precipitated by the addition of 50 μl of 3 M sodium acetate and 1 ml of absolute ethanol. Nucleic acids were recovered by centrifugation at 20,000×*g* for 15 min. The DNA pellet was finally washed three times with 70% ethanol, dried, and resuspended in 100 μl of Tris–EDTA (TE) buffer. DNA extract quantity and quality were measured using a Nanodrop spectrophotometer (Thermo Scientific) prior to storage at − 80 °C.

The 16S rRNA genes, region V4, were PCR amplified using the 515 F (5′-GTGYCAGCMGCCGCGGTAA-3′)/806 R (5′-GGACTACNVGGGTWTCTAAT-3′) primer pair and the following PCR procedure: 30 cycles of 45 s at 95 °C, 60 s at 50 °C and 90 s at 72 °C on a thermocycler. The sequencing of amplicons was performed using Illumina MiSeq technology (paired-end reads, 2 × 250 bp). The obtained data were processed using the quantitative insights into microbial ecology (QIIME 2) software package. All amplicon sequence variants (ASVs) were aligned using MAFFT and used to construct a phylogeny with FastTree 2 [95]. The taxonomy was assigned to each ASV using the ‘classify-sklearn’ command in q2-featureclassifier against the Greengenes reference database (version 13.8). Alpha diversity metrics (richness and Shannon index) and beta diversity metrics (Bray–Curtis) were calculated using the q2-diversity command. The differentially abundant bacterial taxa were determined at the genus or species level using analysis of composition of microbiomes (ANCOM) [96].

### Measurement of intestinal AhR activity

The AhR activity of mouse stool samples was measured using a luciferase reporter assay method, as previously described [40]. Briefly, the H1L1.1c2 cell line, containing a stably integrated dioxin response element-driven firefly luciferase, was seeded in a 96-well plate and stimulated with mouse stool suspensions for 24 h. Luciferase activity was measured using a luminometer, and the results are reported as fold changes based on the luciferase activity of an unstimulated control. All values were normalized on the basis of the cytotoxicity of the samples using the Lactate Dehydrogenase Activity Assay (Promega).

### Fecal metabolic profile using ^1^H-NMR spectroscopy

Faecal aqueous extracts were prepared for NMR analysis as previously described [97, 98]. All ^1^H NMR spectra were obtained on a Bruker DRX-600-Avance NMR spectrometer (Bruker) using the MetaToul-AXIOM metabolomics platform (MetaboHUB), operating at 600.13 MHz for the ^1^H resonance frequency, with an inverse-detection 5-mm H1-C13-N15 cryoprobe attached to a cryoplatform (the preamplifier cooling unit). The resultant spectra were phased, baseline corrected, and manually calibrated to trimethylsilylpropanoic acid (TSP) (δ0.00 ppm) using Mnova NMR (version 9.0; Mestrelab Research S.L.). After removing the region containing the water resonance (δ4.6–5.2 ppm), the spectra were normalized to the probabilistic quotient [99] and aligned [100]. Data were mean-centred and scaled using unit variance scaling, followed by analysis with orthogonal projection on latent structure-discriminant analysis (O-PLS-DA). To identify metabolites responsible for discrimination between the groups, we calculated the O-PLS-DA correlation coefficients (r2) for each variable and back-scaled them into a spectral domain, thus preserving the NMR spectra shapes and the coefficient signs [101, 102]. We filtered the correlation coefficients extracted from significant models, such that correlations were only considered significant if they exceeded the threshold defined by Pearson’s critical correlation coefficient (p < 0.05; |r|> 0.7; for n = 10 per group). For illustration purposes, the area under the curve for significant metabolites was integrated.

### Genotoxic assessment

Cells from intestinal and liver tissues were isolated for genotoxicity determination. Briefly, cells from the intestinal tissue segments (size between 1 and 1.5 cm) from the different test groups were isolated by longitudinally opening the piece of intestine with scissors and scraping the internal surface of intestine using a scalpel blade. The cells were then dissociated by incubation at 37 °C for 30 min in a solution of collagenase and collected in PBS after 5 min of centrifugation and aspiration of the supernatant. For the liver segments (weight between 100 and 200 mg), cells were isolated by cutting the sample into small pieces with a scalpel prior to dissociation by incubation at 37 °C for 30 min in a solution of collagenase. After several steps of manual dissociation and centrifugation, the isolated cells were collected in PBS. To ensure the same number of cells for all the samples, the cells isolated from the colons and livers of different groups were counted with a cell counter to produce a cell suspension at 200,000 cells/ml before being deposited in duplicate in 96-well plates (40,000 cells/well).

The γH2AX genotoxicity assay was performed with the in-cell western (ICW) technique as previously described [103–106]. Briefly, the cells in 96-well plates were fixed with paraformaldehyde (Electron Microscopy Science), permeabilized with 0.1% Triton X-100 (Sigma–Aldrich) in PBS and blocked with MAXblock Blocking Medium (Active Motif) before being washed with PBS supplemented with 2% foetal calf serum and 0.1% Triton X-100 (PST buffer). Cells were then incubated with rabbit monoclonal anti-γH2AX primary antibody (Clone 20E3, Cell Signaling) in PST buffer. After three washes in PST buffer, secondary detection was carried out using an infrared fluorescent dye conjugated to goat antibody (CF770, Biotium). For DNA labelling, RedDot2 (Biotium) labelling was used. After incubation followed by three washes, DNA and the biomarker of interest (γH2AX) were simultaneously visualized using an Odyssey Infrared Imaging Scanner (LiCor ScienceTec, Les Ulis, France). For the determination of genotoxicity, relative fluorescent units for γH2AX per cell (as determined by the γH2AX content divided by the DNA content) were divided by the respective controls (vehicle only) to determine the change in the level of phosphorylation of histone H2AX compared with the control group.

### Statistical analysis

Data visualization and statistical analysis were conducted using GraphPad Prism version 9.0 (GraphPad software, San Diego, Ca, USA). Statistical analysis for the intestinal permeability measurements, intestinal immune response and gut microbiota analysis were conducted as follows:

Normal distribution was verified using the Kolmogorov–Smirnov test with Dallal–Wilkinson–Lillie correction. Statistical differences were determined using one-way analysis of variance (ANOVA) followed by the post hoc Tukey’s test for multiple comparisons. When assumptions of a normal distribution were not met, a nonparametric Kruskal–Wallis test was conducted, followed by a post hoc Dunn’s test. For comparisons between two groups, significant differences were determined using the two-tailed Student’s *t* test or nonparametric Mann–Whitney test. In all determinations, statistical outliers (using the 18 ROUT method) or samples where technical issues were encountered, such as poor RNA quality, poor tissue quality for permeability measurements, or poor histological orientation, were removed from analysis. Specifically, for the analysis of the gut microbiota beta-diversity, PERMANOVA was applied using the distances dataset to compare the centroids and dispersion of the experimental groups. If significant differences were indicated (p value < 0.05), pairwise comparisons were performed to determine the conditions that were significantly different from the control group.

For metabolite production analysis, the area under the curve of metabolites was tested for statistical significance using one-way analysis of variance (ANOVA) followed by Sidak’s post-test for multiple comparisons. Metabolites with both a significant correlation coefficient from the predictive axis on the O-PLS-DA models and a significant p value using univariate statistics were considered significantly different between groups.

## Supplementary Information

**Additional file 1**. Supplementary material, table and figures.

## Data Availability

All relevant data are included in the manuscript and supporting information, and available from the authors upon request.

## Abbreviations

AhR: Aryl hydrocarbon receptor
ANOVA: One-way analysis of variance
BW: Body weight
EDX: Energy-dispersive X-ray
EFSA: European Food Safety Authority
EU: European union
F/B: Firmicutes/Bacteroidetes
FITC: Fluorescein isothiocyanate
Gapdh: Glyceraldehyde 3-phosphate dehydrogenase
IFN: Interferon
IL: Interleukin
Lcn: Lipocalin
MCFA: Medium-chain fatty acids
NMR: Nuclear magnetic resonance
NP: Nanoparticle
OPLS-DA: Orthogonal partial least squares discriminant analysis
Ref: Reference
RT-PCR: Reverse transcriptase Polymerase chain reaction
SCFA: Short-chain fatty acids
TEM: Transmission electron microscopy
TER: Transepithelial resistance
TGF: Transforming growth factor
TNF: Tumor necrosis factors
WGA: Wheat Germ Agglutinin
