# Supplementary material for: A 90-day oral exposure to food-grade gold at relevant human doses impacts the gut microbiota and the local immune system in a sex-dependent manner in mice
Source: Part Fibre Toxicol. 2023 Jul 13;20:27. doi: 10.1186/s12989-023-00539-5 (PMC10339616; doi:10.1186/s12989-023-00539-5)
Supplement: Supplementary file 1 — Additional file 1. Supplementary material, table and figures. [file 12989_2023_539_MOESM1_ESM.docx]

**A 90-day oral exposure to food-grade gold (E175) at human relevant doses impacts the gut microbiota and the local immune system in a sex-dependent manner in mice**

Lauris Evariste^1‡^, Bruno Lamas^1‡^, Sandrine Ellero-Simatos^1^, Laure Khoury^2^, Christel Cartier^1^, Eric Gaultier^1^, Benoit Chassaing^3^, Nicolas Feltin^4^, Laurent Devoille^4^, Georges Favre^4^, Marc Audebert^1,2^, Eric Houdeau^1*^.

^1^Toxalim UMR1331 (Research Centre in Food Toxicology), Toulouse University, INRAE, ENVT, INP-Purpan, UPS, Toulouse, France.

^2^PrediTox, Toulouse, France.

^3^INSERM U1016, team "Mucosal microbiota in chronic inflammatory diseases", CNRS UMR 8104, Université de Paris, Paris, France.

^4^Department of materials, LNE, Trappes, France.

^‡^These authors contributed to this work as co-first authors.

^*^Corresponding author: [eric.houdeau@inrae.fr](mailto:eric.houdeau@inrae.fr)

**Supplementary material**

Table S1: Oral exposure to E175 and Ref-Au. Data are expressed as mean ± SEM

|  | | E175 | | | Ref-Au |
| --- | --- | --- | --- | --- | --- |
|  | Target intake (µg/kg of BW/d) | 0.1 | 1 | 10 | 10 |
| Male | Calculated intake (µg/kg of BW/d) | 0.09 ± 0.002 | 0.93 ± 0.02 | 9.43 ± 0.18 | 9.08 ± 0.17 |
| Female | Calculated intake (µg/kg of BW/d) | 0.11 ± 0.002 | 1.14 ± 0.02 | 11.64 ± 0.19 | 11.85 ± 0.18 |


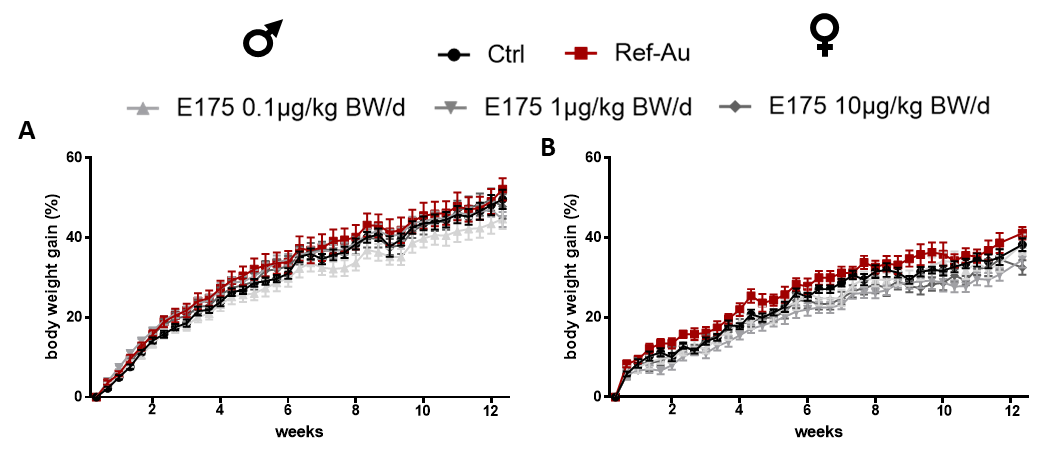


**Figure S1: Body weight gain of male and female mice exposed to Ref-Au or E175 for 13 weeks.** (A) Body weight gain of male mice orally exposed for 13 weeks to Ref-Au at 10µg/kg bw/d or E175 at 0.1, 1 and 10µg/kg bw/d. (B) Body weight gain of female mice orally exposed for 13 weeks to Ref-Au at 10µg/kg bw/d or E175 at 0.1, 1 and 10µg/kg bw/d. The data are expressed as the mean±SEM. *P<0.05 by two-way ANOVA and *post hoc* Bonferroni test.


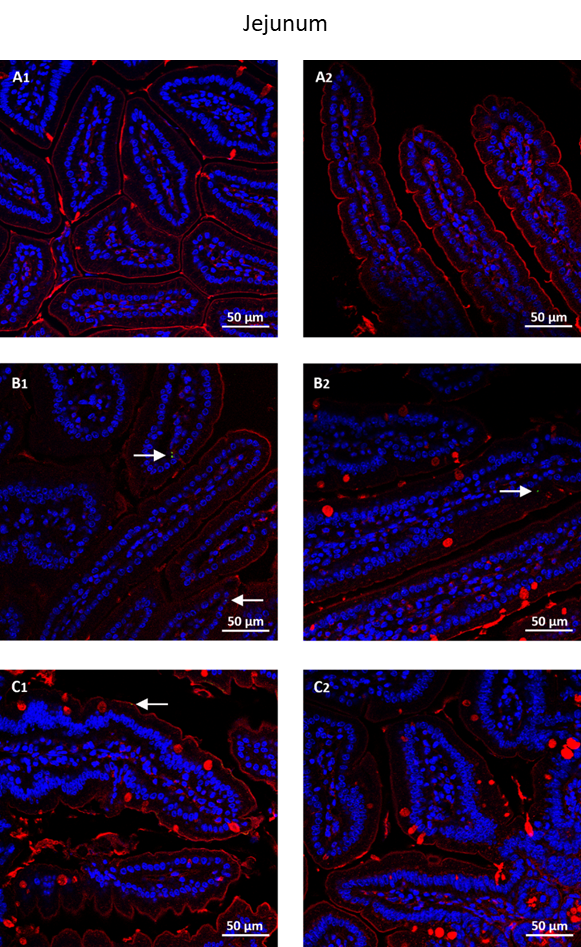


**Figure S2: Confocal images of jejunum tissue sections from mice orally treated for 13 weeks days with gold-containing preparations.** (A) control, (B) Ref-Au nanomaterial, and (C) E175. The laser reflecting (metal) particles appear in green (white arrows), the WGA-labelled glycoproteins in red, and cell nuclei in blue.


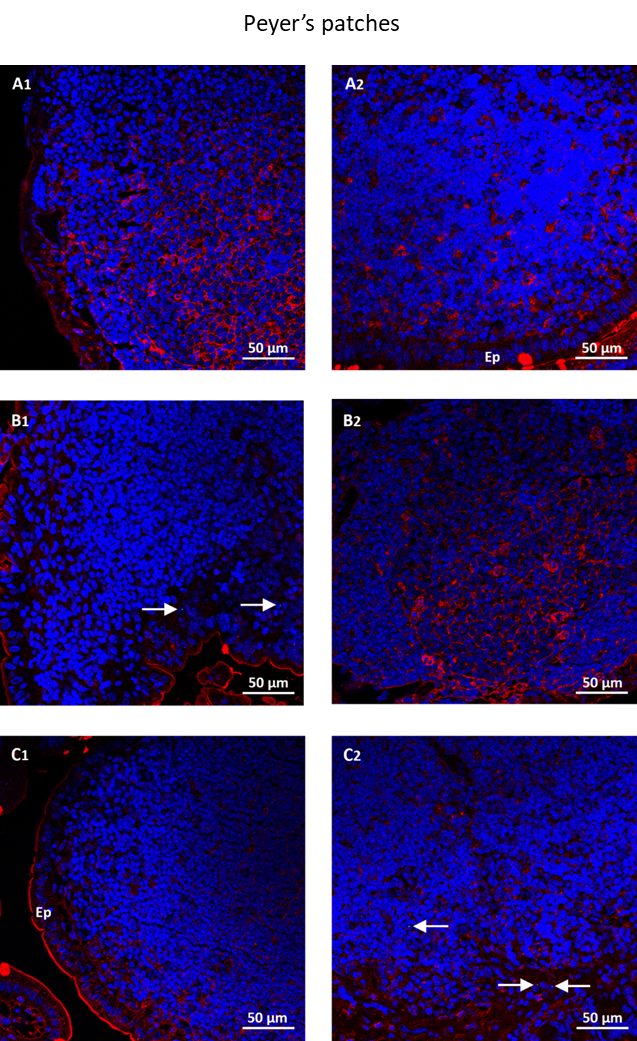


**Figure S3: Confocal images of Peyer’s patches tissue sections from mice orally treated for 13 weeks with gold-containing preparations**. (A) control, (B) Ref-Au nanomaterial, and (C) E175. The laser reflecting (metal) particles appear in green (white arrows), the WGA-labelled glycoproteins in red and cell nuclei in blue. Ep: epithelium.


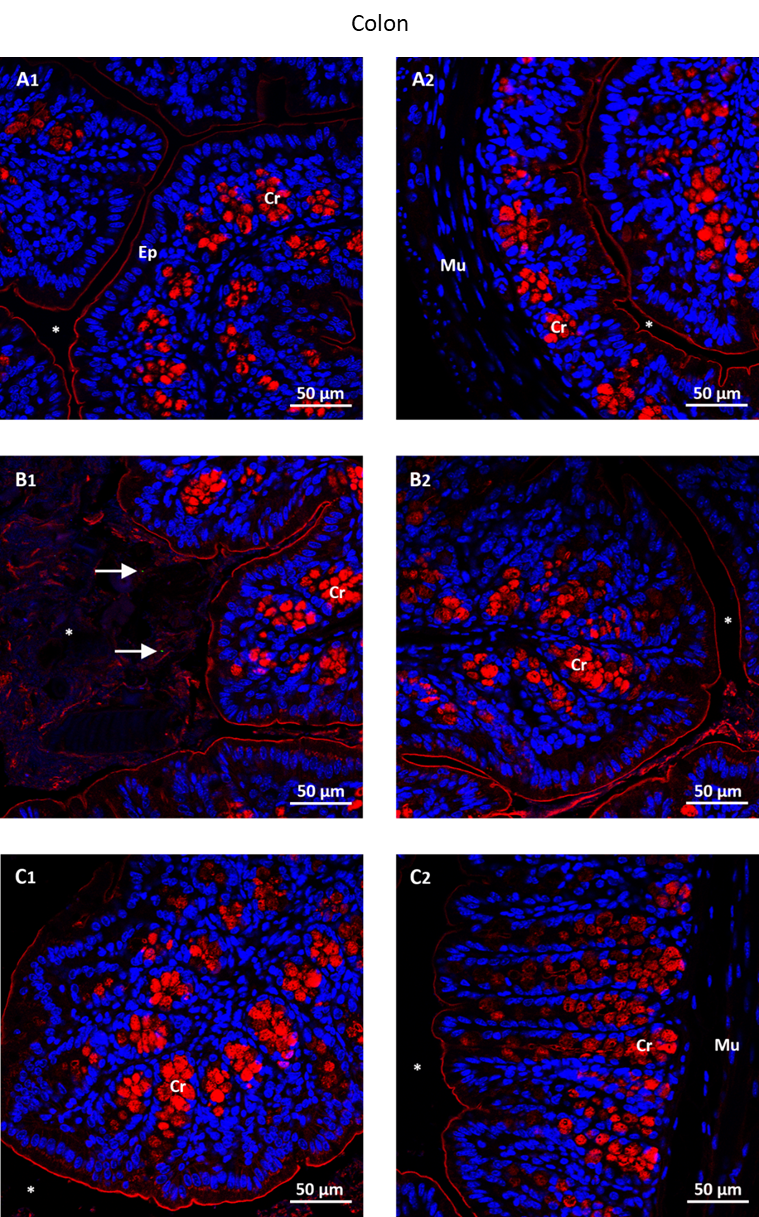


**Figure S4: Confocal images of colon tissue sections from mice orally treated for 13 weeks with gold-containing preparations**. (A) control, (B) Ref-Au nanomaterial, and (C) E175. The laser reflecting (gold) particles appear in green (white arrows), the WGA-labelled glycoproteins in red and cell nuclei in blue. Ep: epithelium; Mu: muscle; Cr: crypt; *: lumen.


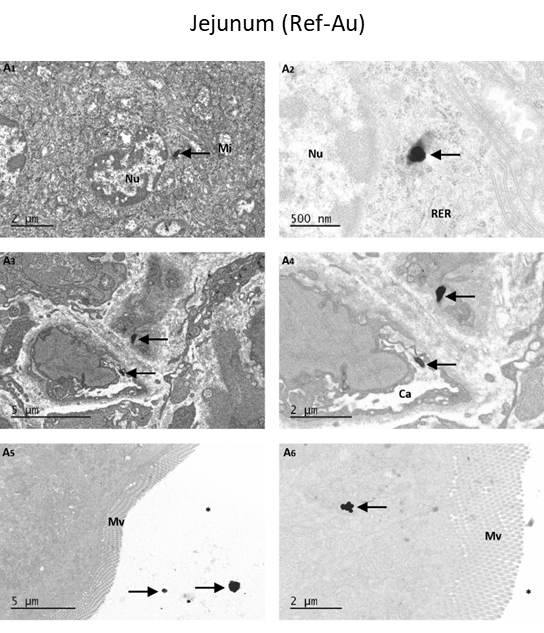


**Figure S5. Representative TEM analysis in the small intestine of mice treated with the Ref-Au nanomaterial.**

**A:** TEM images of tissue sections from the jejunum. Paired right and left panels in A1-A4 correspond to the same tissue sections at different magnification.

*Arrows* indicate electron-dense inorganic particles. Note in A5 some particles distributed into the lumen of the intestine (i.e., unabsorbed).

Nu: nucleus; Mv: microvilli; Ca: capillary; RER:  rough endoplasmic reticulum; *: intestinal lumen.


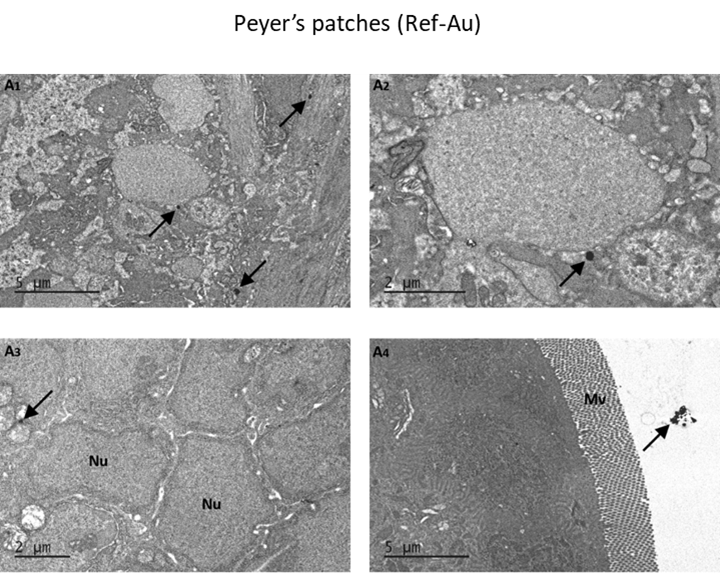


**Figure S6. Representative TEM analysis in the Peyer’s patches of mice treated with the Ref-Au nanomaterial**

**A:** TEM images of Peyer’s patches (gut-associated lymphoid tissue). Paired right and left panels in A1-A2 correspond to the same tissue sections at different magnification. *Arrows* indicate electron-dense inorganic particles.

Nu: nucleus; Mv: microvilli; *: intestinal lumen.


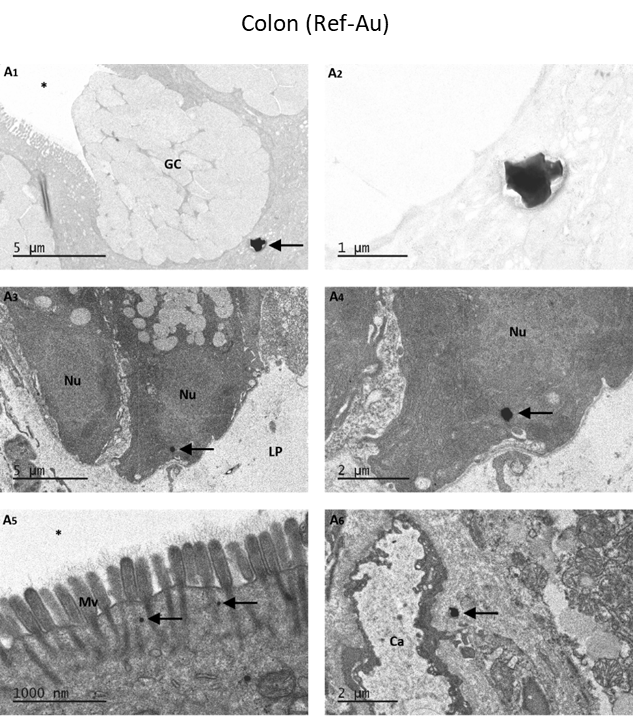


**Figure S7. Representative TEM analysis in the colon of mice treated with the Ref Au nanomaterial**

**A:** TEM images of colonic tissue sections. Paired right and left panels in A1-A4 correspond to the same tissue sections at different magnification. *Arrow*s indicate electron-dense inorganic particles.

GC: Goblet cell; Nu: nucleus; Mv: microvilli; Ca: capillary; *: intestinal lumen.


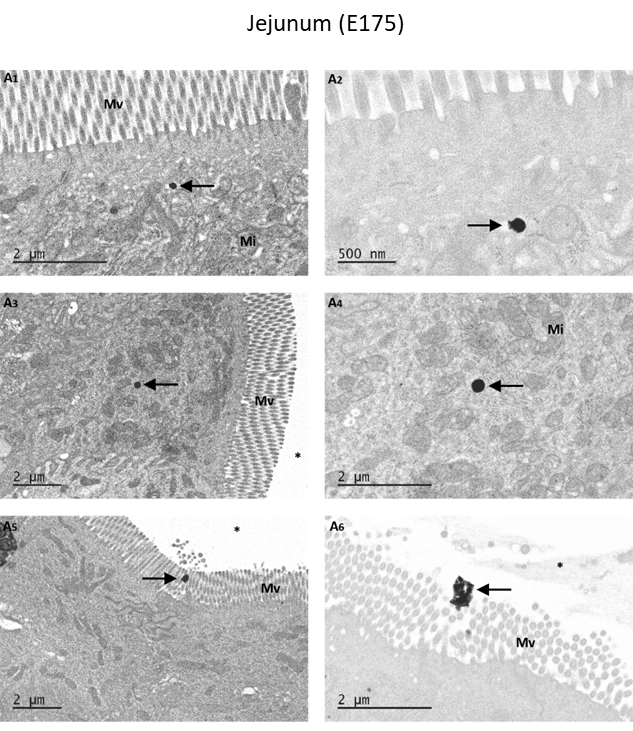


**Figure S8. Representative TEM analysis in the small intestine** **of mice treated with the food additive E175**

**A:** TEM images of tissue sections from the jejunum. Paired right and left panels in A1-A4 correspond to the same tissue sections at different magnification. *Arrows* indicate electron-dense inorganic particles.

Mv: microvilli; Mi: mitochondria; *: intestinal lumen.


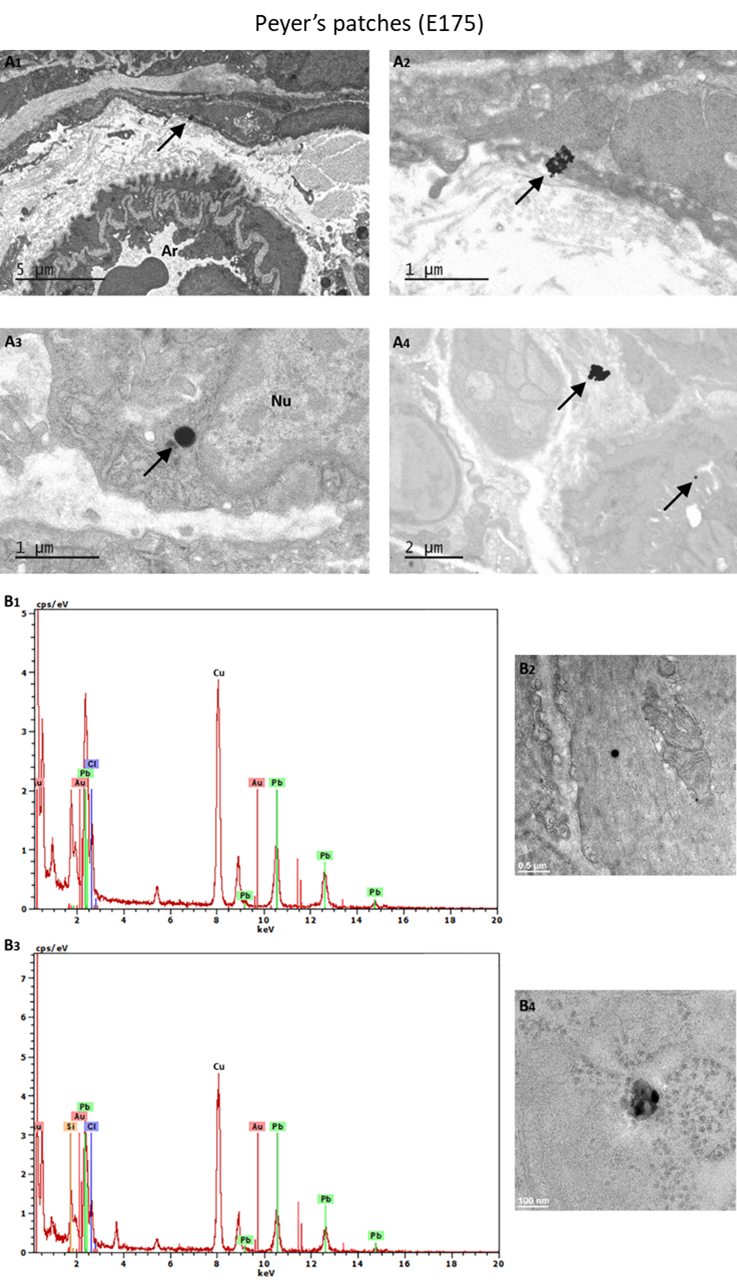


**Figure S9. Representative TEM-EDX analysis in the Peyer’s patches of mice treated with the food additive E175**

**A:** TEM images of Peyer’s patches (gut-associated lymphoid tissue). Paired right and left panels in A1-A2 correspond to the same tissue sections at different magnification. *Arrows* indicate electron-dense inorganic particles.

**B:** representative EDX spectra (B1, B3) and corresponding TEM images (B2, B4).

Nu: nucleus; Ar: Artery.


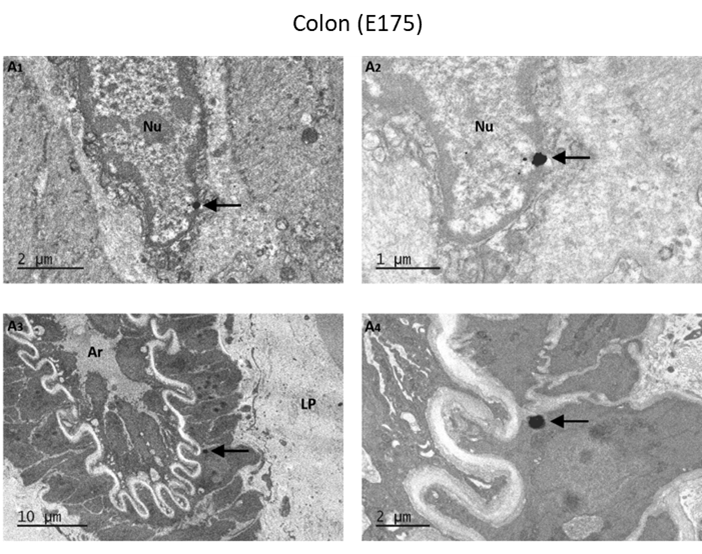


**Figure S10. Representative TEM analysis in the colon of mice treated with the food additive E175**

**A:** TEM images of colonic tissue sections. Paired right and left panels in A1-A2 correspond to the same tissue sections at different magnification. *Arrows* indicate electron-dense inorganic particles.

Nu: nucleus; Mv: microvilli; Ar: artery.


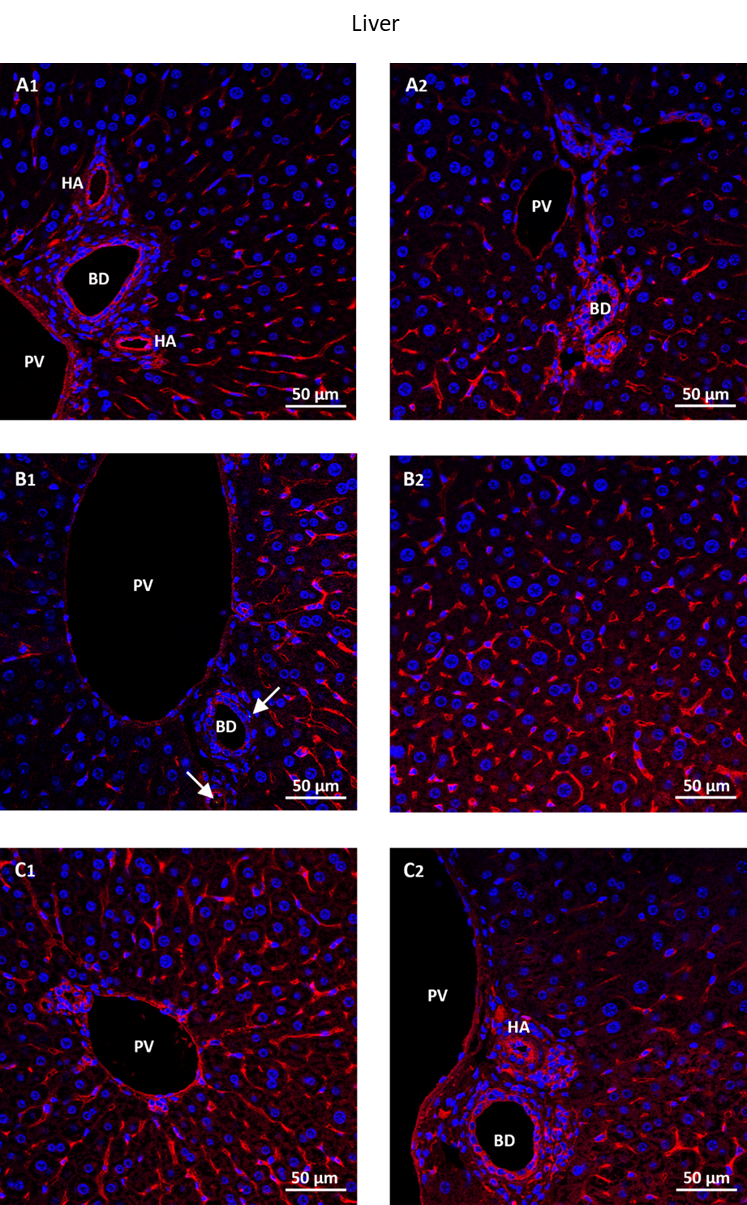


**Figure S11: Confocal images of liver tissue sections from mice orally treated for 13 weeks with gold-containing preparations**. (A) control, (B) Ref-Au nanomaterial, and (C) E175. The laser reflecting (metal) particles appear in green (white arrows), the WGA-labelled glycoproteins in red and cell nuclei in blue. PV: portal vein; BD: bile duct; HA: hepatic artery.


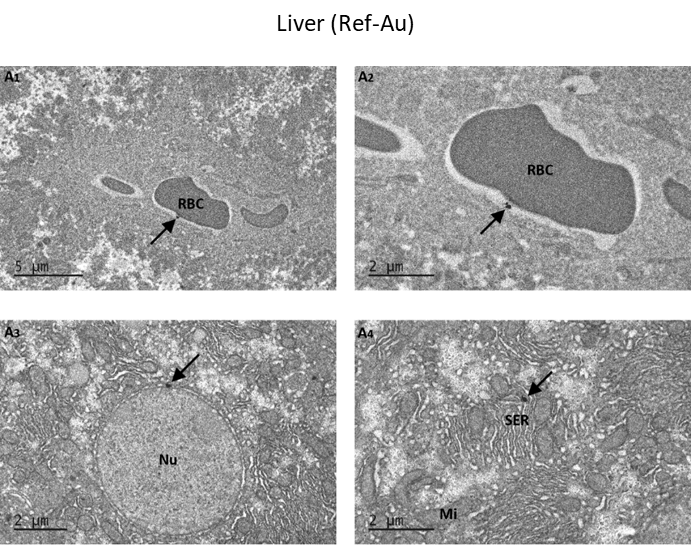


**Figure S12. Representative TEM analysis in the liver of mice treated with the Ref Au nanomaterial**

**A:** TEM images of liver tissue sections. Paired right and left panels in A1-A2 correspond to the same tissue sections at different magnification. *Arrows* indicate electron-dense inorganic particles.

Nu: nucleus; SER : smooth reticulum endoplasmic; RBC: red blood cell.


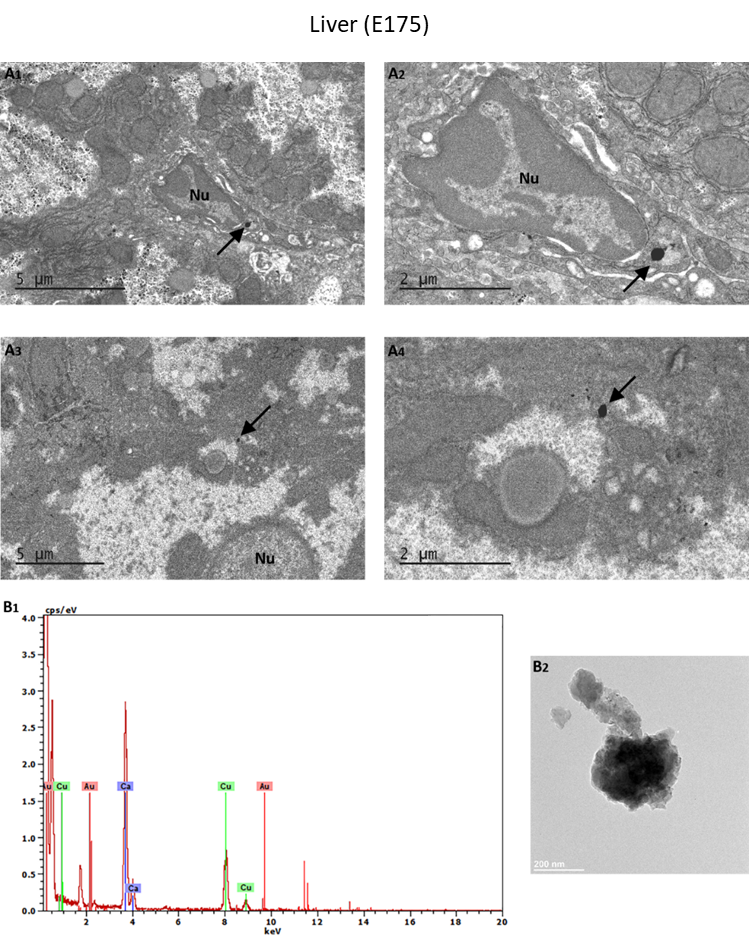


**Figure S13. Representative TEM-EDX analysis in the liver of mice treated with the food additive E175**

**A:** TEM images of liver tissue sections. Paired right and left panels in A1-A2 and A3-A4 correspond to the same tissue sections at different magnification. *Arrows* in A indicate electron-dense inorganic particles.

**B :** representative EDX spectrum (B1) and corresponding TEM image (B2).

Nu: nucleus.


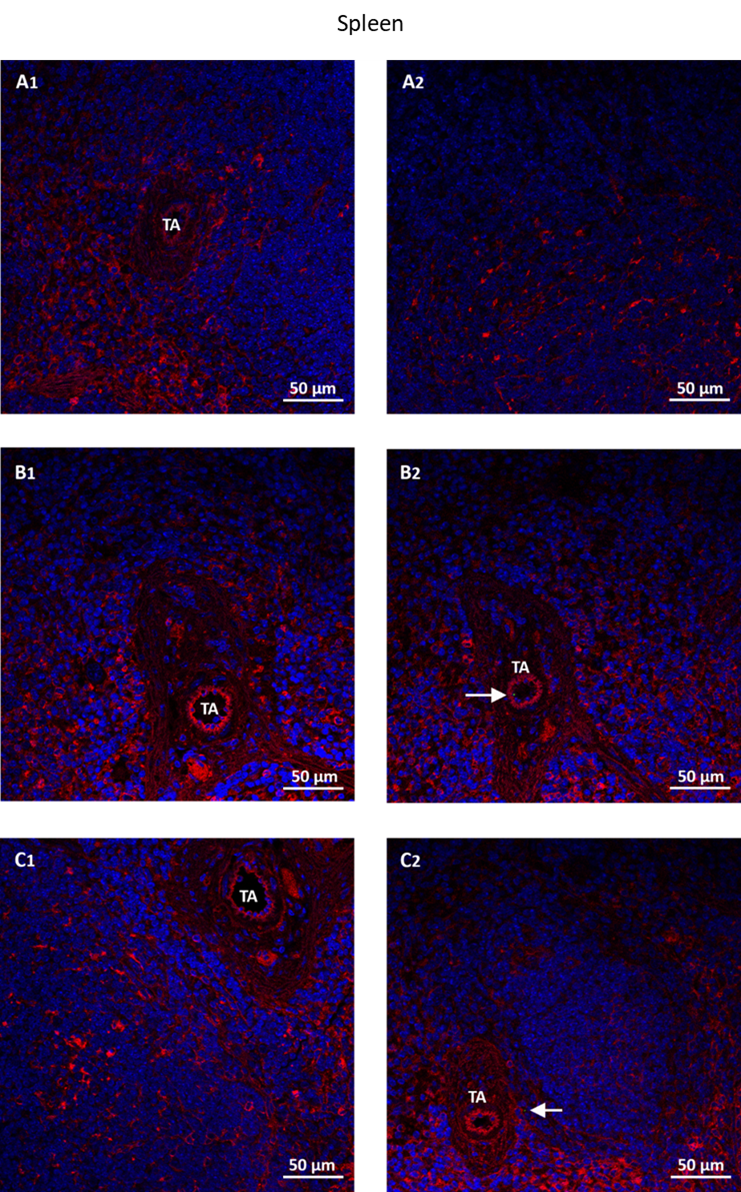


**Figure S14: Confocal images of spleen tissue sections from mice orally treated for 90 days with gold-containing preparations**. (A) control, (B) Ref-Au nanomaterial, and (C) E175. The laser reflecting (metal) particles appear in green (white arrows), the WGA-labelled glycoproteins in red and cell nuclei in blue. TA: trabecular artery.


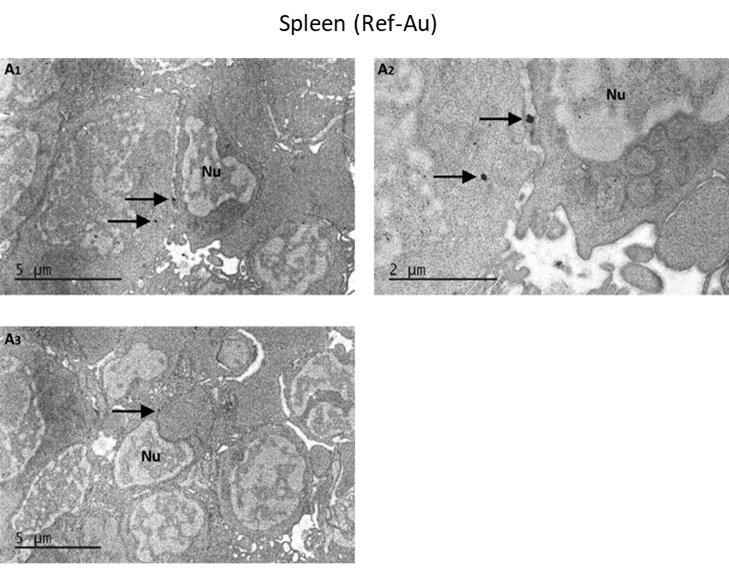


**Figure S15. Representative TEM analysis in the spleen of mice treated with the Ref Au nanomaterial**

**A:** TEM images of spleen tissue sections. Paired right and left panels in A1-A2 correspond to the same tissue sections at different magnification. *Arrows* in A indicate electron-dense inorganic particles.

Nu: nucleus.


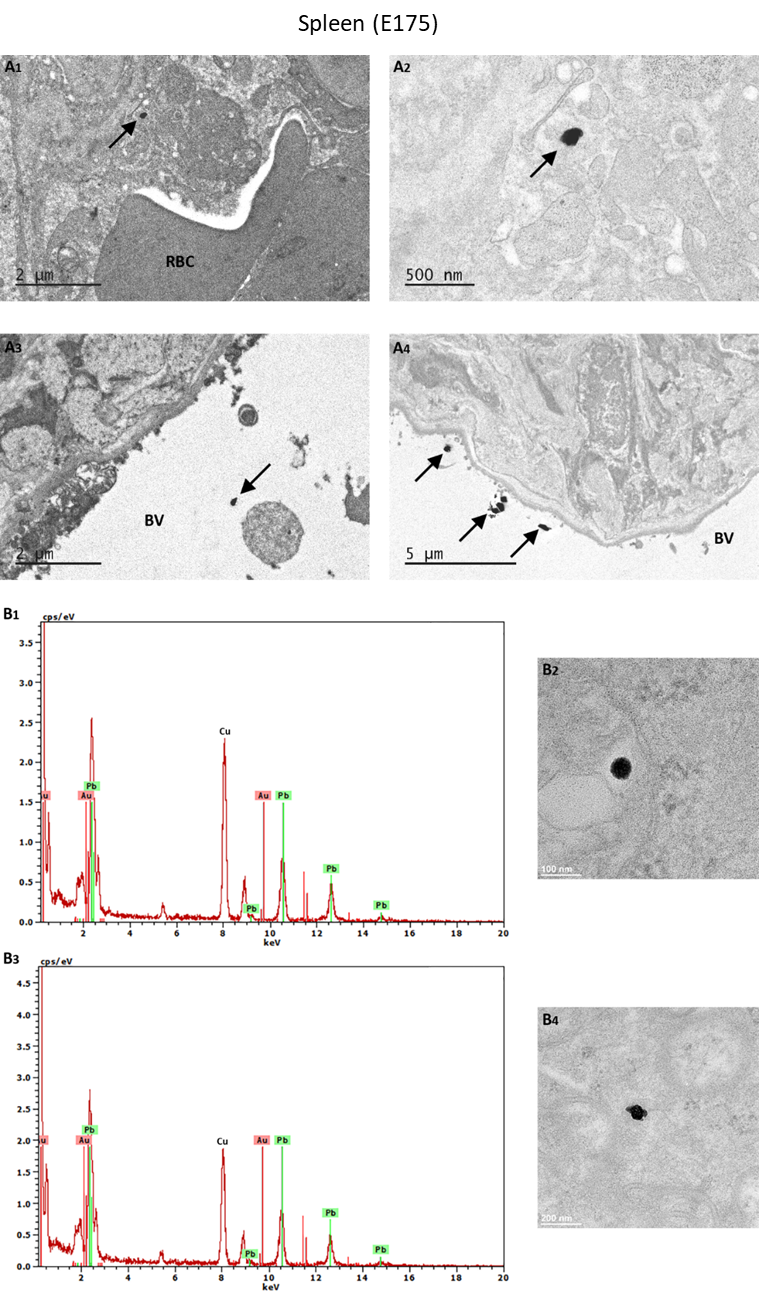


**Figure S16. Representative TEM-EDX analysis in the spleen** **of mice treated with the food additive E175**

**A:** TEM images of spleen tissue sections. Paired right and left panels in A1-A2 correspond to the same tissue sections at different magnification. *Arrows* indicate electron-dense inorganic particles.

**B:** representative EDX spectra (B1, B3) and corresponding TEM images (B2, B4) of EDX-analyzed particles.

RBC: red blood cell; BV: blood vessel.


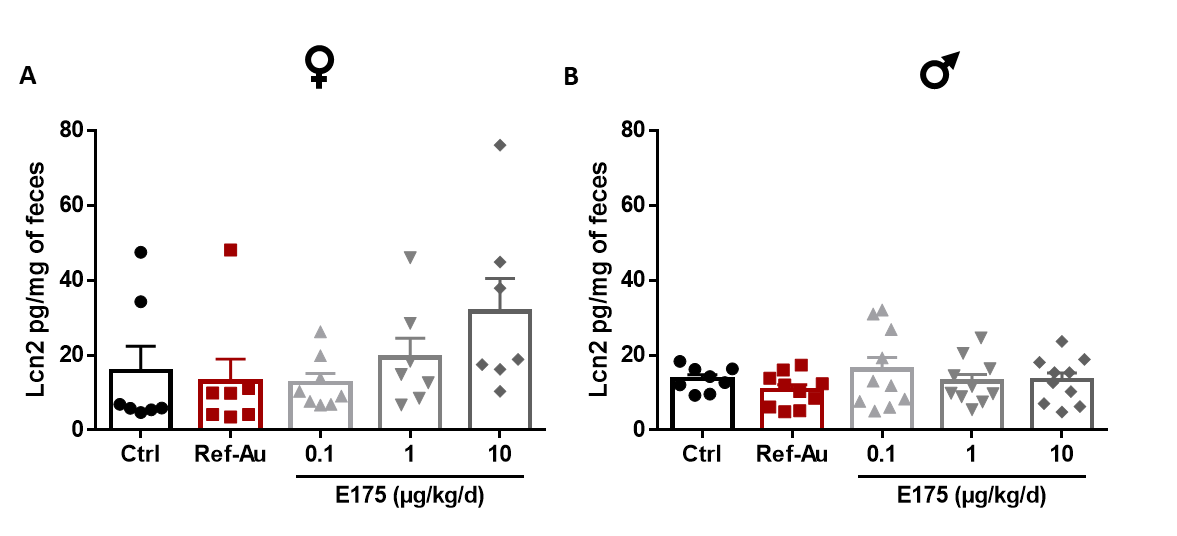


**Figure S17: Lipocalin-2 (Lcn2) levels in feces of female and male mice exposed for 13 weeks to the Ref Au nanomaterial or the E175.** Fecal level of Lcn-2 in female (A) and male (B) mice exposed to exposed to Ref-Au (10µg/kg bw/d) or E175 0,1; 1; 10µg/kg bw/d) for 13 weeks. Each dot represents an individual mouse. Data are presented as mean ± SEM. Statistical significance was determined by one-way ANOVA with Tukey post hoc test; **p*<0.05.


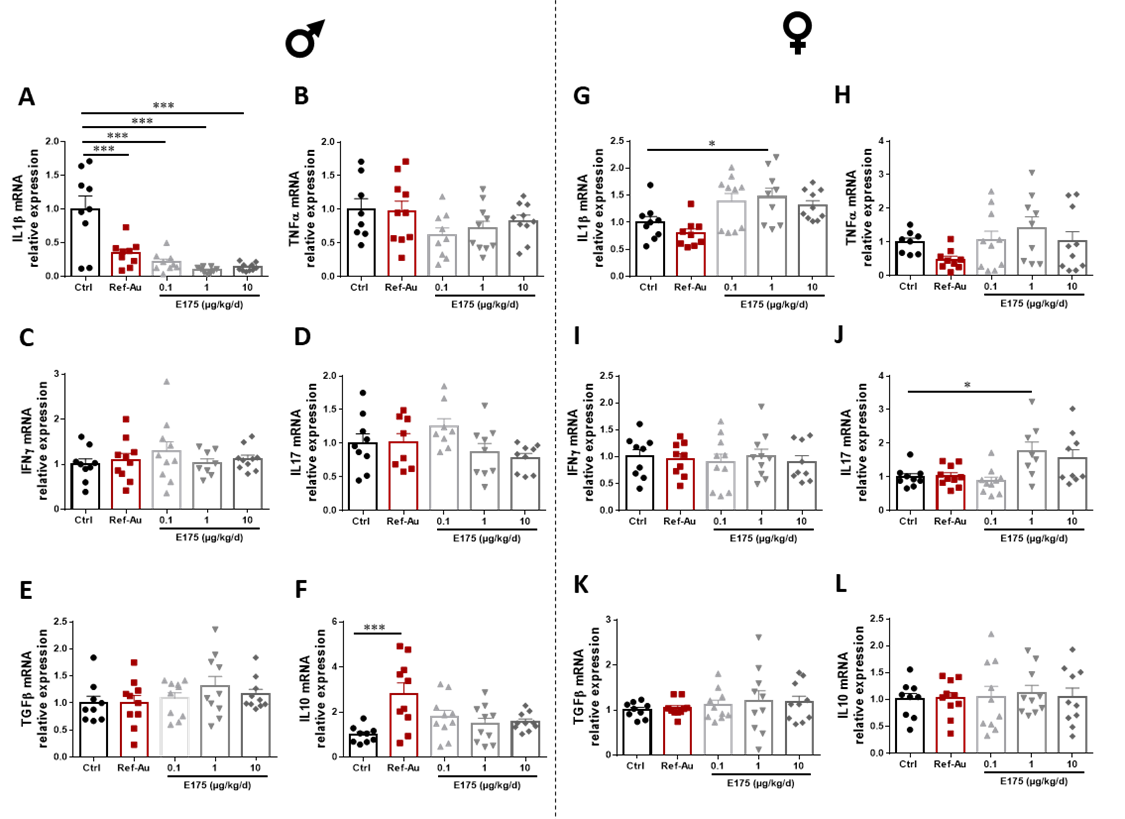


**Figure S18: Gene expression of pro- and anti-inflammatory cytokines in colon of male and female mice exposed to the Ref-Au nanomaterial or E175 for 13 weeks.** Gene expression of the pro-inflammatory cytokines IL1β (A,G), TNFα (B, H), IFNγ (C, I) and IL17 (D,J) as well as of the anti-inflammatory cytokines TGFβ (E, K) and IL10 (F,L) in the colon mucosa of male (A-F) and female (G-L) mice orally exposed to Ref-Au (10µg/kg bw/d) or E175 (0.1; 1 and 10µg/kg bw/d) for 13 weeks. Each dot represents an individual mouse. Data are presented as mean ± SEM. Statistical significance was determined by one-way ANOVA with Tukey post hoc test or Kruskal-Wallis followed by Dunn’s post hoc test; *p<0.05, **p<0.01, ***p<0.001.


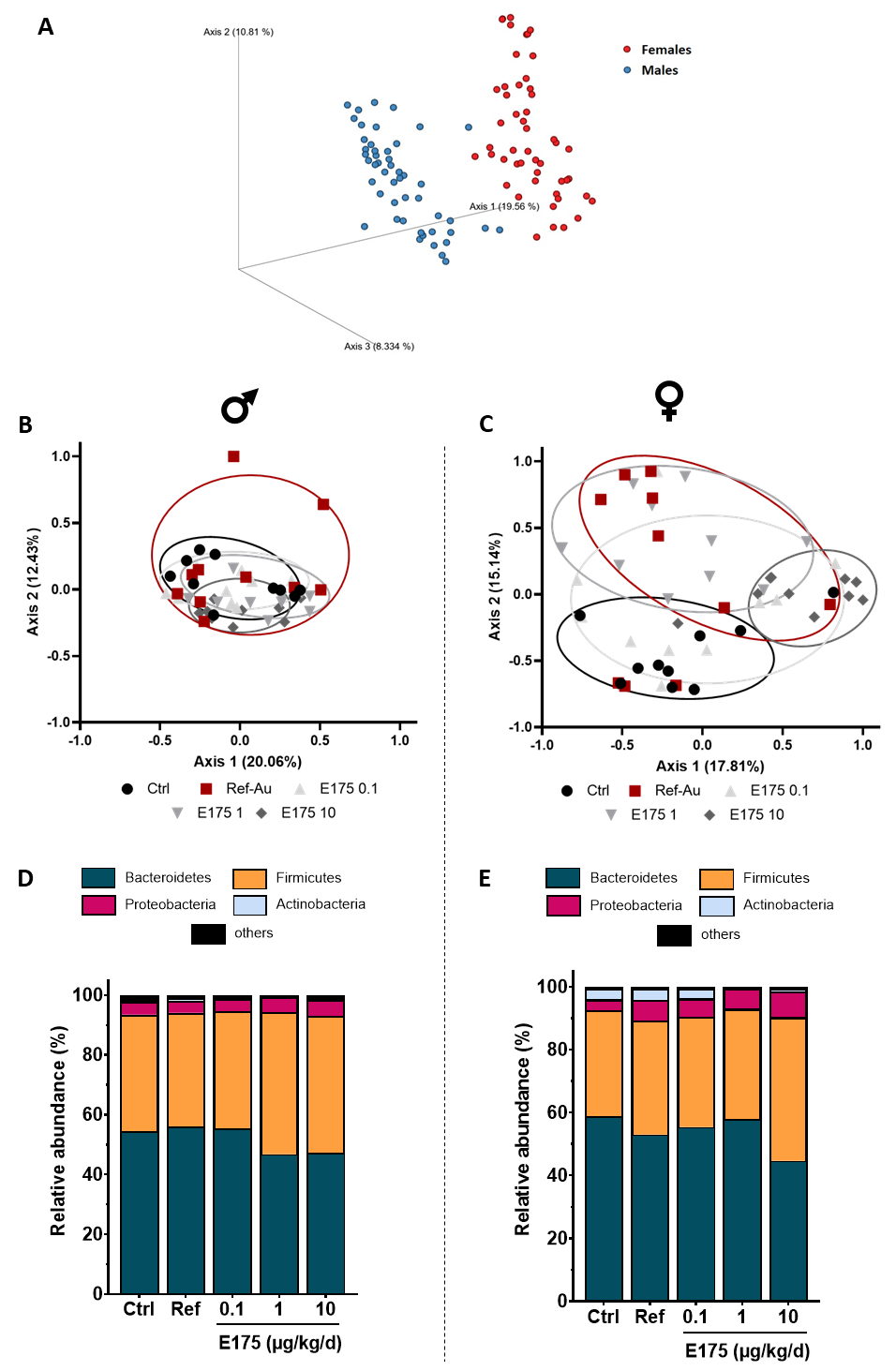


**Figure S19. Sex-specific differences in gut microbiota composition of mice exposed to Ref-Au or E175 for 13 weeks.**

**A:** Principal component analysis based on Bray-Curtis dissimilarity calculated on bacterial 16S rDNA gene sequence abundance in feces from male and female mice exposed to Ref-Au (10µg/kg bw/d) or E175 0,1; 1; 10µg/kg bw/d) for 13 weeks.

**B-C:** Principal component analysis based on Bray-Curtis dissimilarity calculated on bacterial 16S rDNA gene sequence abundance in feces from male (B) or female (C) mice exposed to Ref-Au (10µg/kg bw/d) or E175 0,1; 1; 10µg/kg bw/d) for 13 weeks.

**D-E**: Bacterial-taxon-based analysis at the phylum level in the feces from male (B) and female (C) mice exposed to Ref-Au (10µg/kg bw/d) or E175 0,1; 1; 10µg/kg bw/d) for 13 weeks.


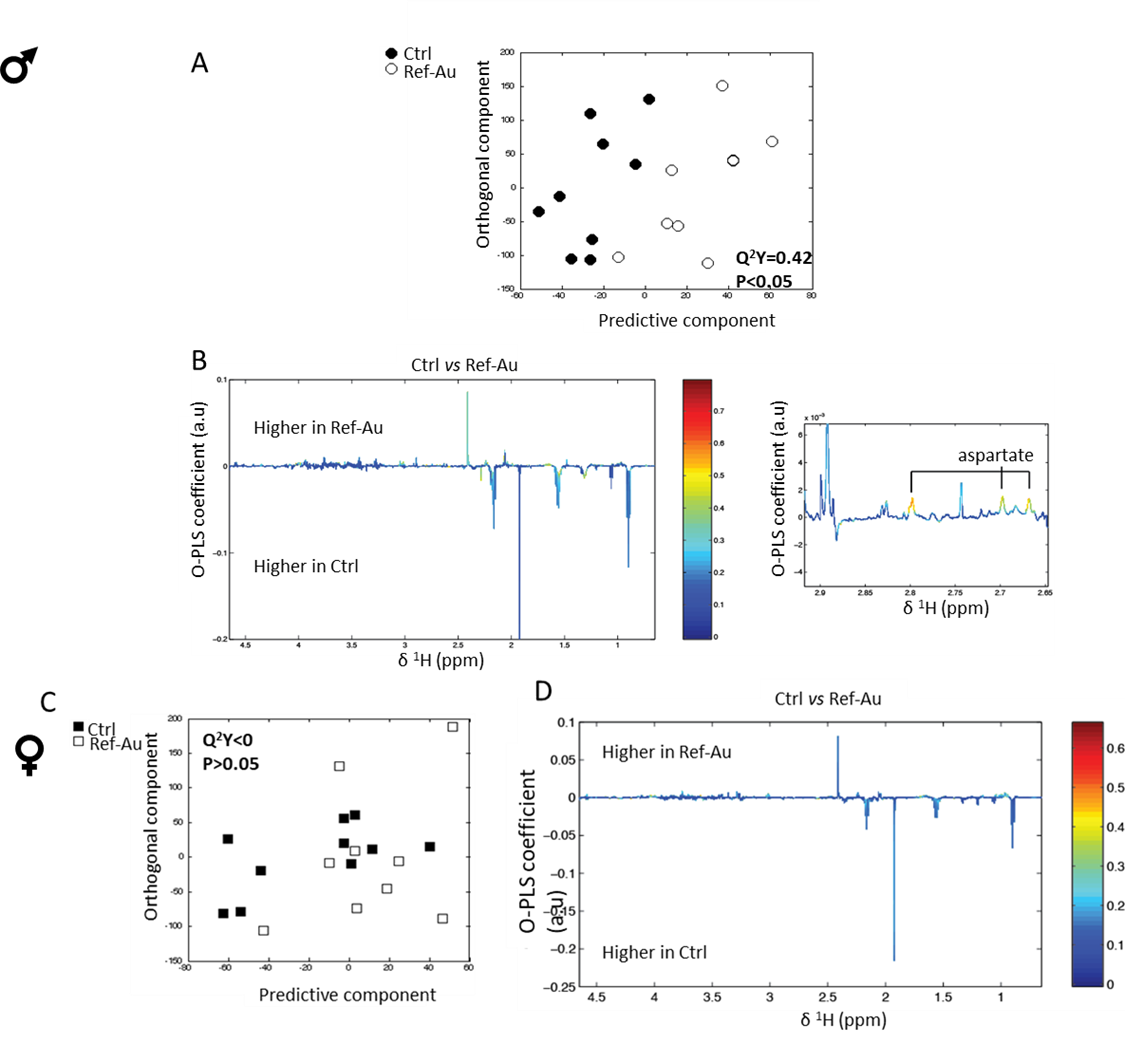


**Figure S20: Effects of exposure to Ref Au nanomaterial on the metabolic activity of the gut microbiota in male and female mice.** (A, C) Orthogonal projection on latent structure-discriminant analysis (O-PLS-DA) score plots derived from ^1^H-NMR spectra of fecal extract from males (A) and female (C) exposed to the Ref Au nanomaterial (Ref-Au) and mice exposed to untreated food pellets (Ctrl). (B,D) Coefficient plots related to the O-PLS-DA models discriminating between males (B) or female (D) mice exposed to the Ref Au nanomaterial (Ref-Au) and mice exposed to untreated food pellets (Ctrl). Panels in (B) and (D) show the discriminant metabolites that are higher or lower in mice exposed to the Ref Au nanomaterial compared to mice exposed to untreated food pellets. Metabolites are color-coded according to their correlation coefficient, red indicating a very strong positive correlation. The direction of the metabolite indicates the group with which it is positively associated as labeled on the diagram
